# Learning-based orchestration for low-latency AI deployment in hybrid cloud–edge platforms

**DOI:** 10.1038/s41598-026-58531-w

**Published:** 2026-06-20

**Authors:** Ahmed Albugmi

**Affiliations:** Computer and Information Technology Department, Applied College, King Abdulaziz University, 80213 Jeddah, Saudi Arabia

**Keywords:** Distributed AI, Deep learning deployment, Resource-aware scheduling, MLPerf benchmark, Inference optimization, Cloud–edge computing, Model placement, Adaptive orchestration, Engineering, Mathematics and computing, Computer science, Information technology, Software

## Abstract

The rapid growth of AI-driven applications in hybrid cloud–edge environments poses substantial challenges to ensuring low latency, high throughput, and effective resource utilization. Conventional deployment models, which are typically fixed or policy-driven, are not sufficiently flexible to respond dynamically to changing workloads and heterogeneous hardware environments. In this work, we introduce and analyze a resource-conscious deep learning-based scheduling system for managing the deployment of AI models on distributed cloud edges. The framework improves inference performance by leveraging real-time system telemetry and model features generated by benchmarks, while maintaining quality of service (QoS) compliance. The proposed system uses a fully connected neural network trained on structured features derived from the MLPerf Inference Benchmark, including compute complexity, memory footprint, and input dimensions. It is guided by real-time data from a hybrid infrastructure (NVIDIA A100/V100 GPUs and Jetson Xavier edge devices) to inform scheduling. Four MLPerf inference workloads – ResNet 50, BERT, SSD ResNet34, and DLRM – were tested and contrasted across various batch sizes and latency thresholds. Generalization experiments with unseen models such as GPT 2 and YOLOv5 yielded > 90% success rates in deployment, with the latency reduction and throughput gain results as presented above. Results of the generalization experiments with unseen models, including GPT 2 and YOLOv5, demonstrated deployment success rates > 90% for the various profiling conditions evaluated, with the latency reduction and throughput improvements as shown above. The results show that learning-based orchestration can be used to deliver space- and resource-aware orchestration solutions that are adaptive for low-latency deployment of AI services in hybrid cloud edge systems, but the effectiveness of the solution will depend on the representativeness of the profiling data and similarity of training and deployment environments.

## Introduction

AI-powered applications are based on large-scale distributed systems comprising cloud data centers, edge clusters, and hybrid environments for deploying large-scale models. Optimizing the use of computing, memory, and network resources to provide low-latency inference and high throughput is a major challenge. As deep learning models become more complex, the challenges of efficient deployment and management increase^1^. The traditional methods of AI implementation are exemplified by tools such as Kubernetes^2^ and TensorFlow Serving^3^. They are more likely to use rigidly defined or manual scheduling rules that, in most cases, cannot be modified to accommodate variability in resource availability or workload. MLPerf Inference established a model where the performance of inference is calculated, and so complex deployment strategies are required^4^. The efficiency improvements have led to the creation of resource-conscious deployment systems that scale to the existing compute and model needs^5^. However, the current mechanisms are unable to adapt to a changing workload profile or system performance. This means that current distributed AI systems lack effective, data-driven methods for intelligently positioning and scheduling models. Existing AI implementation strategies are typically incapable of handling distributed systems with heterogeneous resource availability and workloads^6^. Rushed changes and improvised resource control are likely to result in waste, suboptimal performance, or additional expenditure. It must have a flexible deployment system capable of assigning resources on the fly based on workload characteristics, model preferences, and system feedback.

This paper seeks to address the challenge of developing a deep learning solution that can handle resource constraints during model implementation. The objective is to improve the performance of AI using machine learning to analyze the performance measurements derived based on benchmarks like MLPerf and modify deployment strategies to ensure a high inference effectiveness with low latency and contention.

As AI models become more intricate and bigger, the effective implementation of these models on a heterogeneous hardware set is currently a major issue. Inference of high performance demands us to utilize state-of-the-art hardware and rational use of CPU, memory, and bandwidth. The conventional approaches, e.g., the use of static containers or manually tuned workloads, are usually unable to match the caprice of cloud and edge environments^2^. Moreover, the growing range of hardware platforms used to execute AI systems (e.g., CPUs, GPUs, TPUs, and edge accelerators) makes the resource management task more complex^7^. Unadjusted policies can also cause suboptimal use of resources or significant variation in the latency of variable environments^8^. Inference workflows can attempt to trade off the throughput, responsiveness, and resource consumption when the workflow becomes large, and results that conventional deployment systems fail to achieve optimally^9^. A promising solution for scheduling has been offered by emerging methods based on machine learning. Deep learning makes it possible for system behavior and workload patterns to be modeled, providing for real-time adaptive deployment decisions. With standardized workloads and performance benchmarks on the MLPerf Inference Benchmark, we can effectively train deep learning models for deployment^10,11^. This research is motivated by the need to close the gap between static deployment approaches in the conventional world and the dynamic, performance-sensitive needs of AI inference in a distributed world. The present work addresses the development of a solution for AI model deployment in distributed environments using a deep learning-based scheduling method. The specific objectives are:

- To develop a resource-aware deployment framework that leverages deep learning for adaptive model placement across heterogeneous cloud and edge nodes.
- To design a feature extraction and training methodology using MLPerf Inference Benchmark data for predicting optimal deployment decisions under real-time constraints.
- To evaluate the proposed system against baseline schedulers regarding latency, throughput, and resource utilization using real-world infrastructure and standardized workloads.

To examine the framework’s scalability, generalizability, and scheduling overhead under controlled hybrid cloud edge testbed conditions, thereby assessing its potential applicability to larger AI inference deployments. Placement decisions in heterogeneous cloud–edge deployment can only be made online in the face of dynamic telemetry, queueing effects, and a variety of hard constraints (e.g., GPU memory and SLA latency). Optimization solvers may yield good solutions to small or static problems, but re-solving a constrained problem at runtime can be expensive, and they are not scalable when requests are sporadic and significantly change the system state. Because of this reason, we use a machine-learning-based policy that is trained offline based on profiling and past results and generates near-linear-time placement decisions at run-time. This paper proposes a deep learning-based framework for resource-aware model deployment to accelerate AI inference workflows in distributed systems. The key contributions of this work are as follows:

- A novel deployment framework that integrates system-level resource monitoring with model performance profiling to enable adaptive AI inference in heterogeneous distributed environments.
- A deep learning model trained using features derived from the MLPerf Inference Benchmark, capable of predicting high utility deployment decisions under varying workload and hardware conditions while respecting resource and latency constraints.
- An evaluation pipeline that benchmarks our system against baseline schedulers (e.g., static allocation, heuristic policies), demonstrating improvements in inference latency, throughput, and resource utilization.
- A reproducible implementation of our framework and training setup, including annotated MLPerf-derived features and deployment scripts, made available to the research community for further development and validation.

The contributions are intended to fill the gap between the traditional deployment strategies and performance-conscious and intelligent orchestration in current distributed AI infrastructures.

The remainder of this paper is organized as follows: The related Work section reviews distributed AI deployment, resource-aware scheduling, and system-level optimization. The system design section presents the general system architecture and the deployment framework design. The methodology section describes the approach, which comprises problem formulation, feature engineering, deep learning model architecture, experimental setup, workloads, and baseline methods used in the evaluation. The results and evaluation section presents analyses of latency, throughput, and utilization. The discussion section talks about the essential findings, generalizations, and limitations. Lastly, the conclusion and future work section concludes this paper and outlines directions for future work.

## Related work

Scaling AI workloads across distributed systems depends on efficient resource management. These systems need to support a variety of compute nodes, heterogeneous equipment, and variable network topology, and be subject to hard performance and cost requirements. It is further complicated by the dynamic nature of AI workloads, which frequently involve bursts of inference requests or variable compute demand when deploying models. The classic cluster managers include Borg^12^, Kubernetes^2^, and Mesos^13^, with most having a static or rule-based scheduler that assigns resources according to constraints or priorities that are preset. Although they have been shown to be useful with general-purpose workloads, when used on AI tasks, deep learning inference, they are typically ineffective because they do not understand the performance properties of models and hardware sensitivities. More intelligent scheduling methodologies have been studied recently. In one case, Gandiva^14^ proposed a fine-grained distributed system for sharing GPUs in DNN training, where cluster throughput is enhanced by adjusting to model behavior. Likewise, Tiresias and SLAQ^15^ have committed to workload scheduling for deep learning on a fair, priority-sensitive basis in a multi-tenant environment. Tiresias, a GPU cluster manager for distributed deep learning scheduling, demonstrates that workload-specific cluster managers can achieve shorter completion times than general-purpose systems^15^. Equally, SLAQ aims to optimize the overall quality of multi-tenant machine learning jobs through resource-aware scheduling^16^. Resource heterogeneity and the dynamism of workload demands are additional problems in distributed systems. TensorFlow Serving^3,17^, TorchServe, ONNX Runtime, and TorchServe are the most widely used deployment tools, and are all interested in model packaging to run the models in different backends. Although these frameworks facilitate batching, quantization, and targeting devices, the common feature of these frameworks is that they use fixed settings that fail to change dynamically with changes in the system load or device availability. In more developed systems, deployment is combined with orchestration systems, such as Kubernetes. These configurations are based on autoscaling, container placement, and node affinity rules to manage deployments^2^. Nevertheless, these heuristics are coarse-grained and can not see model-specific performance profiles, including the behavior of a ResNet or BERT model at a particular GPU with different batch sizes. Numerous studies have tried to integrate resource profiling into the deployment choice^18^. For instance, InferLine^19^ uses a multi-stage profiling and optimization pipeline to deliver latency targets by controlling batch sizes and hardware selection. SageMaker Multi-Model Endpoints^20^ implement model multiplexing to improve deployment efficiency, but are still limited by rule-based resource allocation. Despite these advances, few systems dynamically learn from runtime feedback to improve deployment over time. The majority of them are based on offline profiling or a set of rules, and they do not generalize to different scenarios of deployment and different hardware conditions. This disparity is driving us to work and we introduce a deep learning based deployment plan that can infer the best maps of resource assignments in real time system measurements and model workload characteristics. Deep learning has recently been applied to modeling and prediction tasks and optimizing complex system-level operations. In distributed computing, these methods have shown promise for improving scheduling, load balancing, resource prediction, and task placement, traditionally handled by heuristic or rule-based algorithms. One significant advantage of deep learning is its ability to capture non-linear relationships and high-dimensional patterns in system telemetry and workload behavior. In particular, reinforcement learning (RL) approaches have successfully solved dynamic resource management problems, such as cluster scheduling and task placement. Interestingly, the DeepRM system implemented by Google^21^ used deep reinforcement learning to schedule jobs in the data centers, and it was better than the old system of heuristic scheduling because of the ability to adapt to changes in workload. Supervised deep learning has been applied to forecast resource uses or choices of deployment settings. Indicatively, AutoTVM^22^ is an example of a model that employs neural networks to forecast the best implementations of kernels on hardware backends. Likewise, MorphNet^23^ uses deep learning to optimize neural network designs for runtime efficiency, given a set of resource constraints, the final step between model design and deployment optimization. Within the area of AI inference, studies have concentrated on the prediction of bottlenecks of performance and the latency sensitivity of deep models. As an example, CherryPick^24^ uses Bayesian optimization and learning models to find the best cloud settings to run ML applications. Most of these methods are, however, designed to address a situation that is static or when training workloads are addressed, and in most cases presuppose that the pre-profiling data is easily accessible. We are also different because we train a deep model using dynamic deployment features based on standardized benchmarks (MLPerf) in a way that allows making real-time, predictive deployment decisions. This design is flexible when it comes to hardware and model variations to improve the scalability and coordination of AI inference in a distributed setting. The benchmarking of AI systems is the first step in performance evaluation, which gives a substantial understanding of the effective functioning and development of the systems. Standard benchmarks are necessary to provide objective measurements of model inference tasks and provide the necessary optimizations at the system level. Owing to its development by MLCommons^25^, the MLPerf Inference Benchmark has become a common benchmark used to measure the performance of machine learning models on a large variety of hardware and deployment environments. MLPerf is an evaluation of real-world AI models on real-world image classification (ResNet-50), object detection (SSD-ResNet34), natural language tasks (BERT), recommendation engines (DLRM), and similar tasks. Different types of testing are used on models, such as single-stream, multi-stream, and server environment, and therefore the benchmark can be applied to both edge and datacenter environments^26^. The benchmark presents important data to use during deployment planning with its metrics, which include: latency, throughput, and power efficiency. Although it is helpful in the evaluation, MLPerf has wider purposes for the research community. The standardized versions of workloads, input sizes, and references of pre-trained models allow the researchers to reliably determine performance features of modeling. A number of papers have used MLPerf data to understand hardware behavior, scaling trends, and inference bottlenecks^4,27^. As Table 1 shows, although the number of systems (e.g., InferLine, SageMaker) that deal with the inference deployment is not the lowest, the majority of them are based on predefined rules or fixed profiles. Methods that are training-based (e.g., DeepRM, MorphNet) are able to be flexibly applied but do not deal with latency-sensitive inferences. By contrast, the suggested framework also stands out as having a unique capability of supporting real-time, predictive deployment decisions based on learning of dynamically changing workload features and system telemetry, facilitated by interaction with MLPerf.

**Table 1:** Summary of related work on resource-aware AI deployment in distributed systems.

System/Method	Target Task	Scheduler Type	Adaptability	Strengths	Limitations
Kubernetes^2^	General	Rule-based	Low	Widely adopted, supports autoscaling	No model-awareness, coarse-grained heuristics
TensorFlow Serving^3^	Inference	Static	Low	Supports batching, quantization	Lacks dynamic adaptation
Mesos^13^	General	Rule-based	Low	Fine-grained resource sharing	Not tailored for ML workloads
Gandiva^14^	Training	GPU-aware sharing	Medium	Fine-grained GPU slicing	Not designed for inference workloads
InferLine^19^	Inference	Profile-based pipeline	Medium	Latency-aware scheduling with profiling	Limited adaptability to runtime variability
SageMaker Endpoints^20^	Inference	Rule-based multiplexing	Low	Efficient model sharing	Rule-driven, lacks learning-based optimization
DeepRM^21^	Training	Deep Reinforcement RL	High	Adapts to job patterns	Focused on batch workloads
AutoTVM^22^	Training/Optimization	Supervised Learning	Medium	Predicts kernel-level optimization	Not workload-aware at runtime
MorphNet^23^	Training	Architecture optimizer	Medium	Resource-efficient model adaptation	Static design-time use
CherryPick^24^	Training/Deployment	Bayesian Optimization	Medium	Identifies cloud instance configurations	Offline and profiling-intensive
MLPerf Inference^25^	Inference Benchmark	N/A	N/A	Rich, standardized performance dataset	Used for benchmarking, not for deployment decisions

In this work, we use MLPerf as the foundation for training and evaluation. By extracting structured data from benchmark runs—including input type, batch size, latency, and device characteristics, we enable our deep learning model to learn deployment strategies that are generalizable and grounded in realistic, diverse workload profiles.

## System design

### Overview of deployment framework

**Fig. 1:**
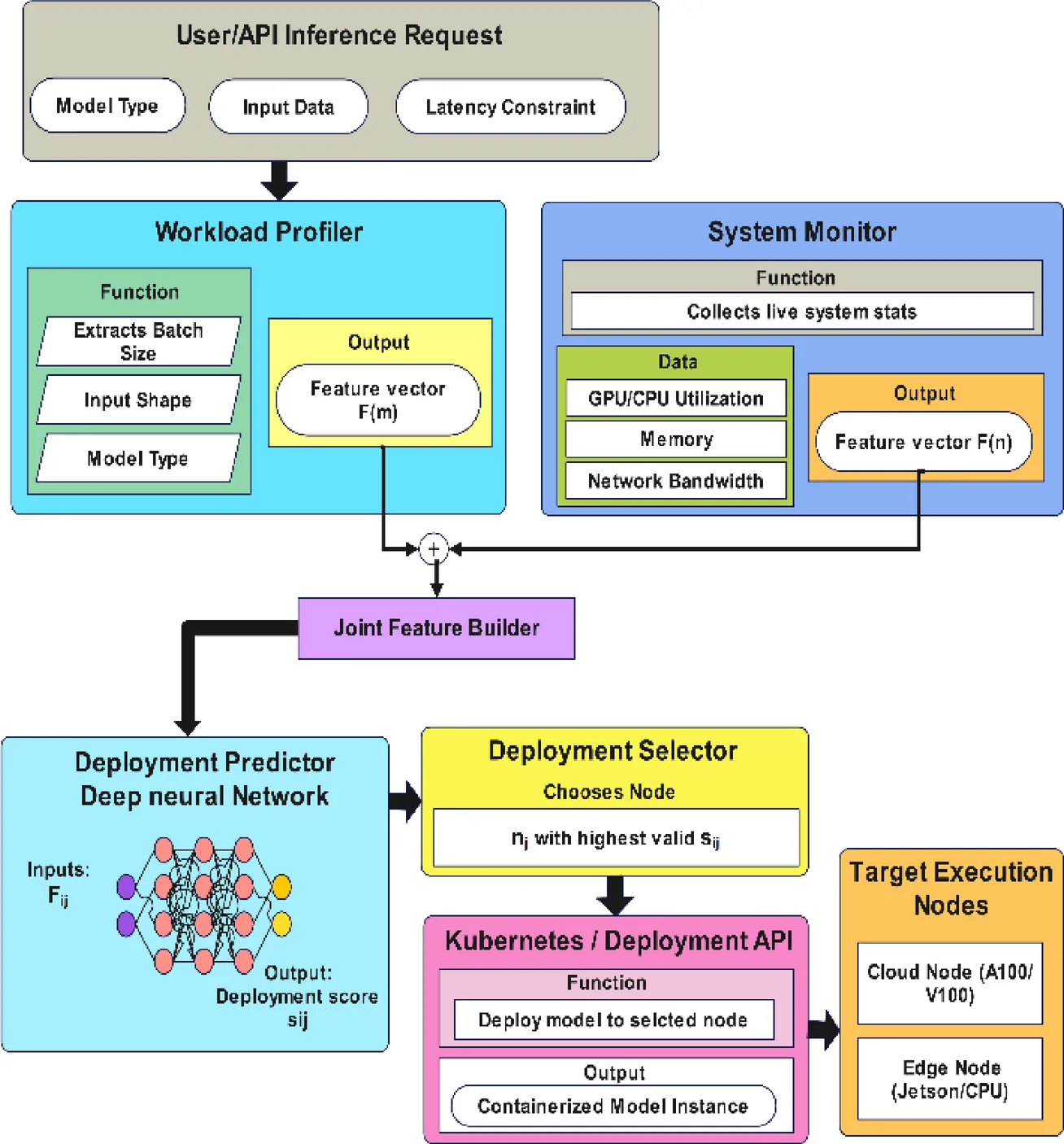
System architecture of the proposed resource-aware model deployment framework.

The proposed framework is designed to automate and optimize model deployment for AI inference in distributed environments. It leverages deep learning to make resource-aware deployment decisions, informed by real-time system telemetry and benchmark-derived workload profiles. On a high-level, the suggested framework consists of four main parts, namely the Workload Profiler, which reads input features and latency limits of the inference requests; the System Monitor which collects real-time resource metrics of the distributed nodes; the Deployment Predictor, a deep neural network that trains on features of the MLPerf-based feature and predicts optimal deployment strategies; and the Orchestration Engine, which integrates with systems such as Kubernetes to execute the deployment strategies in real-time. These modules enable adaptive, resource-aware model deployment across heterogeneous cloud and edge environments, as shown in Fig. 1.

### Resource-aware scheduling mechanism

To operate deep learning models effectively in a distributed system, we develop a resource-conscious scheduling algorithm that selects the best compute node, model type, and configuration (e.g., batch size) based on the model’s needs and the system’s current resource state. This research aims to minimize inference latency while maximizing hardware utilization in dynamic environments. Our scheduler uses a lightweight policy based on a deep neural network that maps the current state of the system to node scores, routing incoming model demands to candidate nodes in a heterogeneous cluster. As an input, the scheduling policy receives the feature representation based on two sources, offline profiling and online telemetry. Information on offline profiling captures model-specific attributes like expected latency and resource usage of various types of devices, whereas the online telemetry information indicates the state of the system in situ, such as the GPU usage, available memory, and queueing status. The scheduler at runtime first eliminates infeasible nodes that are in violation of hard resource or latency constraints. It then chooses the node with the highest predicted score using the trained policy model and the remaining candidate nodes. The model output is then a ranking or score of the list of feasible candidate nodes, on which the ultimate deployment decision is based. When we are training, the scheduler is developed as an instructed learning problem. The model is conditioned to make deployment choices with the aid of labeled examples based on profiling and scheduling data, minimizing loss on cross-entropy between the assignment output and the desired one. The actual procedure of the runtime scheduling based on the trained model to make decisions regarding the deployment is described in Algorithm 1.

**Algorithm 1:**
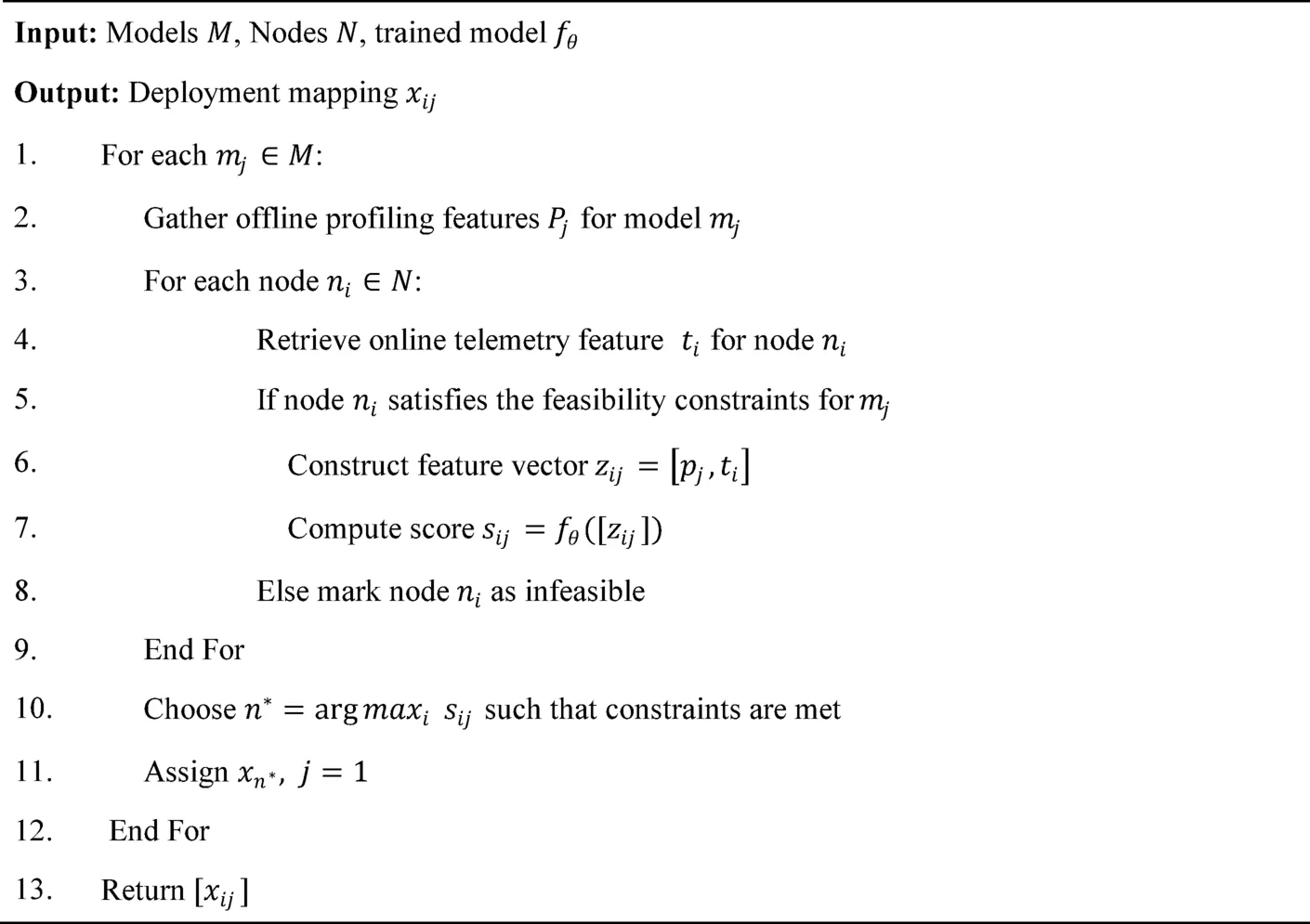
Resource-aware scheduling.

The algorithm is a linear-time scheduling algorithm in terms of the number of nodes and models, and with extension to batching, it would allow simultaneous inference. It enables informed deployment decision-making in a short time without having to tune rules and thresholds manually.

### Scheduler integration and runtime control loop

The proposed scheduler is deployed as an external scheduling service that liaises with the cluster manager and inference serving layer to make placement decisions for incoming requests under heterogeneous resource constraints. At runtime, node-level metrics (e.g., queueing metrics, physical memory availability, GPU utilization) are continuously collected by monitoring agents and transmitted to the monitoring server. The scheduler will intermittently obtain the most recent telemetry and integrate it with offline profiling statistics for each model and hardware type to produce the feature vector, which it relies on to position the model.

The runtime control loop operates as follows. (1) The scheduler stores telemetry and updates the telemetry with a fixed frequency. (2) When a request for metadata is received as an inference request, the gateway forwards this request to the scheduler. (3) The scheduler filters the infeasible nodes that have hard constraints (e.g., memory and device availability). (4) The policy model rates the remaining viable nodes exploiting the constructed feature vector. The scheduler selects the node of the best score, which is feasible, and makes the placement decision known to a cluster manager or a gateway. (6) The request is submitted to the selected node to be implemented, and the measured latency and SLA value are recorded to be examined and possibly retrained. This description of the integration clearly allows the scheduler to be inserted into the stack and used to create a repeatable control loop. The telemetry stream to aid communication-aware scheduling, besides the compute-side metrics, which are the utilization of the GPUs and availability of memory, there are also communication-sensitive measures, such as data transfer rate, queueing delay, and link-related bandwidth. The features are added to the predictor input to ensure that it uses placement choices based on the end-to-end state of the existing cloud–edge system instead of merely the unprocessed compute capability of each node.

### Integration with distributed environments

**Fig. 2:**
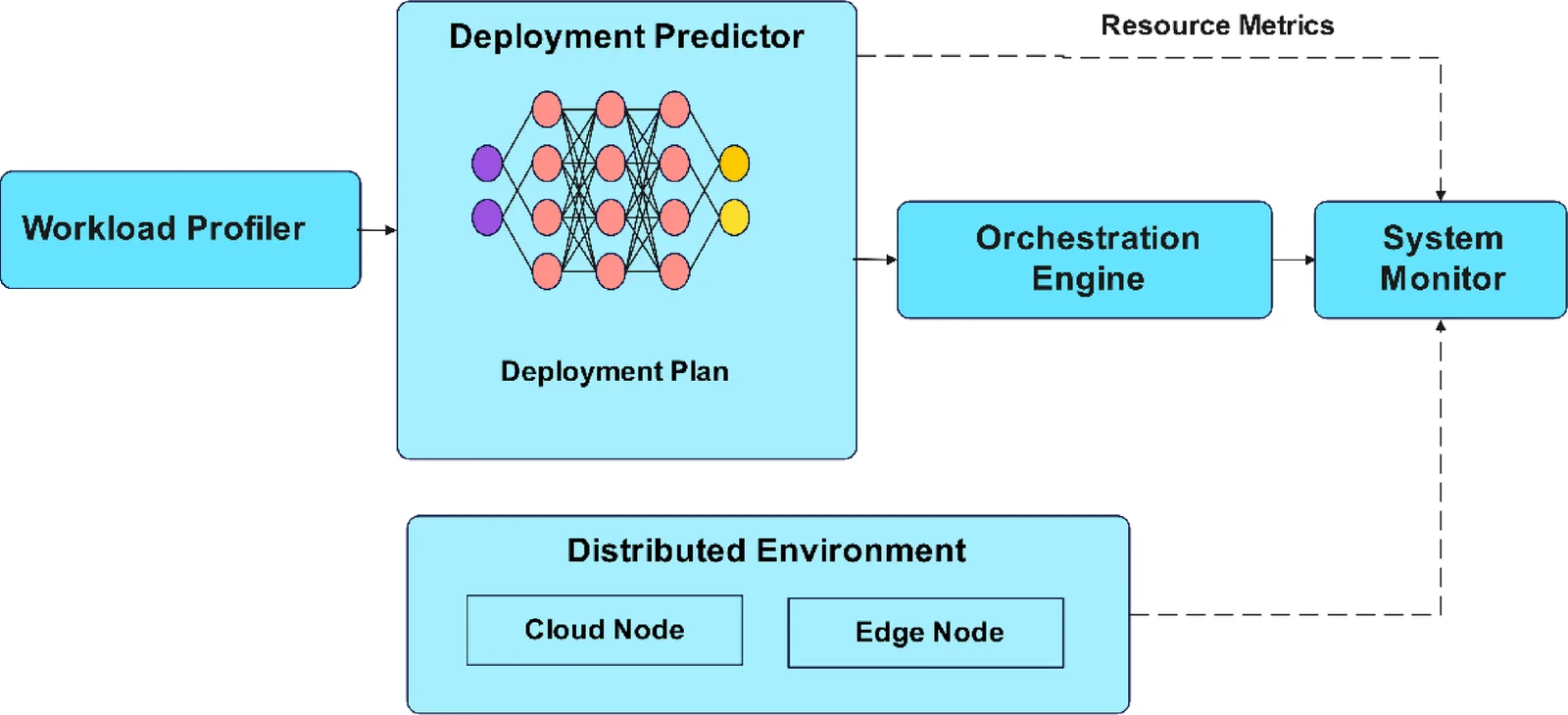
Integration of the resource-aware deployment framework.

To incorporate the proposed deployment framework into distributed systems, the framework’s core components and the heterogeneous components of the infrastructure, such as cloud servers and edge nodes, interact in a coordinated manner. The framework enables modularity, scalability, and real-time adaptability to dynamic situations, designed to work in container ecosystems that can be run by systems like Kubernetes. It begins with the Workload Profiler, which reads metadata from incoming requests for inference workloads. The Deployment Predictor receives the necessary information on the choice of a model, input parameters, and latency requirements and makes a decision. Simultaneously, the System Monitor displays real-time values for every node, such as the overall CPU efficiency, memory usage, data transfer speed, and processor load. The Deployment Predictor is a deep learning model that uses metadata and metrics to generate a deployment plan. The plan provides a description of the optimal node, the device model, the parallelism model, and the batch model that best suit the current situation. After the plan is generated, the orchestration engine performs it on the distributed infrastructure. When cloud nodes offer additional compute capacity necessary for high-throughput scenarios, deployment is on those nodes. For cases that required minimal latency, the System Monitor might redirect the deployment to neighboring edge nodes. The orchestrator manages the deployment by provisioning containers, allocating the required resources, and launching the inference service based on the Deployment Predictor’s decision. After the model is deployed, the System Monitor monitors response time and resource consumption metrics. These metrics are periodically reintroduced into the Deployment Predictor to update or enhance the decision-making model, thereby establishing a feedback-based optimization loop. The system will be able to deploy models in other scenarios through continuous adaptation and resource-oriented decisions, and continue to work even though the environment changes. The roles represented in Fig. 2, Workload Profiler, Deployment Predictor, Orchestration Engine, and System Monitor, are shown to interact with each other, thus allowing the deployment of dynamically resource-sensitive models in various nodes.

This integration ensures that AI inference can be intelligently distributed across compute resources and dynamically adapt to changing workloads, system state, and performance goals. It enables efficient utilization of centralized and decentralized infrastructure and reduces overhead and the inflexibility of rule-based deployment strategies.

## Methodology

### Problem formulation

We address the issue of resource-conscious deployment of a model in a distributed environment that consists of heterogeneous compute nodes. Table 2 presents the summary of the notations used in the problem formulation.

**Table 2:** Notation summary.

Symbol	Meaning
$\:\mathcal{M}$	Set of models
$\:\mathcal{N}$	Set of compute nodes
$\:{x}_{ij}$	Binary decision variable indicating whether model $\:{m}_{i}$ is assigned to node $\:{n}_{j}$
$\:{R}_{j}$	Capacity vector of node $\:{n}_{j}$
$\:{D}_{ij}$	Resource demand vector of model $\:{m}_{i}$ on node $\:{n}_{j}$
$\:{\mathcal{l}}_{ij}$	Estimated inference latency of model $\:{m}_{i}$ on node $\:{n}_{j}$
$\:{\lambda\:}_{i}$	SLA latency threshold for model $\:{m}_{i}$
$\:{u}_{ij}$	Utility score of assigning model $\:{m}_{i}$ to node $\:{n}_{j}$

Let

1 $$\:\mathcal{M}=\{{m}_{1},{m}_{2},\dots\:,{m}_{N}\}$$

denote the set of deep learning models to be deployed, and let

2 $$\:\mathcal{N}=\{{n}_{1},{n}_{2},\dots\:,{n}_{K}\}$$

denote the set of available compute nodes, including both cloud and edge devices.

For each node $\:{n}_{j}\in\:\mathcal{N}$, let

3 $$\:{R}_{j}=\left[{r}_{j}^{\left(1\right)},{r}_{j}^{\left(2\right)},\dots\:,{r}_{j}^{\left(d\right)}\right]\in\:{\mathbb{R}}_{+}^{d}$$

be its resource capacity vector, where * d* is the number of resource types.

For each model-node pair $\:\left({m}_{i},{n}_{j}\right)$, let

4 $$\:{D}_{ij}=\left[{d}_{ij}^{\left(1\right)},{d}_{ij}^{\left(2\right)},\dots\:,{d}_{ij}^{\left(d\right)}\right]\in\:{\mathbb{R}}_{+}^{d}$$

denote the resource demand vector of the model $\:{m}_{i}$ when deployed on a node $\:{n}_{j}$. These demands may vary across nodes due to differences in hardware characteristics and accelerator support.

Each model $\:{m}_{i}$ is also associated with a latency threshold

5 $$\:{\lambda\:}_{i}\in\:{\mathbb{R}}_{+}$$

which represents the maximum acceptable inference delay, and a batch size $\:{b}_{i}$, which may affect both resource usage and latency.

We define $\:{u}_{ij}$ as the utility score of the assigning model $\:{m}_{i}$ to $\:{n}_{j}$. This utility may be used to indicate the quality of an assignment based on latency, QoS satisfaction, or the overall benefit of deployment. In order to prevent confusion, $\:{u}_{ij}$ is employed consistently in the paper to refer to utility, and utilization is used only to refer to the current load of a node and is considered a state of the system, not the optimization objective.

Let

6 $$\:{x}_{ij}\in\:\left\{\mathrm{0,1}\right\}$$

be the binary decision variable defined as

7 $$\:{x}_{ij}=\left\{\begin{array}{cc}1,&\:\mathrm{if\:model\:}{m}_{i}\mathrm{\:is\:assigned\:to\:node\:}{n}_{j},\\\:0,&\:\mathrm{otherwise.}\end{array}\right.$$

The objective is to maximize the total system utility subject to assignment, capacity, and latency constraints:

8 $$\:\underset{{x}_{ij}}{\mathrm{m}\mathrm{a}\mathrm{x}}\sum\:_{i=1}^{N}\sum\:_{j=1}^{K}{x}_{ij}{\hspace{0.17em}}{u}_{ij}$$

Each model is assigned to exactly one node:

9 $$\:\sum\:_{j=1}^{K}{x}_{ij}=1,\forall\:i\in\:\{1,\dots\:,N\}$$

For each node, the total allocated demand must not exceed its available capacity for every resource type:

10 $$\:\sum\:_{i=1}^{N}{x}_{ij}{\hspace{0.17em}}{d}_{ij}^{\left(k\right)}\le\:{r}_{j}^{\left(k\right)},\forall\:j\in\:\{1,\dots\:,K\},\text{}\forall\:k\in\:\{1,\dots\:,d\}$$

Only assignments that satisfy the latency requirement are allowed:

11 $$\:{x}_{ij}{\hspace{0.17em}}{\mathcal{l}}_{ij}\le\:{x}_{ij}{\hspace{0.17em}}{\lambda\:}_{i},\forall\:i\in\:\{1,\dots\:,N\},\text{}\forall\:j\in\:\{1,\dots\:,K\}$$

The latency $\:{\mathcal{l}}_{ij}$depends on both the model and the target node. We define it as

12 $$\:{\mathcal{l}}_{ij}=L({m}_{i},{n}_{j},{b}_{i})={\alpha\:}_{i}\left({b}_{i}\right)\cdot\:\frac{1}{{\mathrm{Throughput}}_{j}}+{\beta\:}_{ij}$$

where:

- $\:{\alpha\:}_{i}\left({b}_{i}\right)$ denotes the effective computational workload of the model $\:{m}_{i}$under batch size $\:{b}_{i}$, measured in FLOPs,
- $\:{\mathrm{Throughput}}_{j}$ denotes the effective compute throughput of the node $\:{n}_{j}$,
- $\:{\beta\:}_{ij}$ represents additional latency components, such as data transfer and queueing delay.

If desired, the utility score $\:{u}_{ij}$ may be defined as a decreasing function of latency, for example:

13 $$\:{u}_{ij}=-{\mathcal{l}}_{ij}$$

or by another QoS-aware scoring rule depending on the scheduling objective.

With collaborative cloud–edge inference, the end-to-end latency depends on both the computational performance and transmission-related factors, including link bandwidth, transmission delay, and inter-tier queues. Thus, the latency model expressly takes into account these communication effects by the name $\:{\beta\:}_{ij}$, which quantifies data transfer time, network-induced delay, and queuing latency involved in the assignment of the model $\:{m}_{i}$ to node $\:{n}_{j}$. This will enable the scheduler to account for compute- and communication-side bottlenecks when evaluating candidate deployments.

This function can be approximated using historical inference logs (e.g., MLPerf) or learned by a performance prediction model. The optimization problem described above is combinatorial and NP-hard due to its binary decision variables and non-linear utility functions. Therefore, solving it exactly at runtime is infeasible for large-scale systems. Instead, we approximate the solution using a trained deep neural network $\:{f}_{\theta\:}(\cdot\:)$, which maps features of $\:({m}_{i},{n}_{j})$ pairs to deployment scores:

14 $$\:{s}_{ij}={f}_{\theta\:}\left({F}_{ij}\right)where\:{F}_{ij}=[{W}_{i},{R}_{j},{\widehat{l}}_{ij}]$$

The deployment decision becomes:

15 $$\:{x}_{ij}=\left\{\begin{array}{c}1,\:\:if\:j=arg{max}_{k}{s}_{ik}\:and\:constraints\:satisfied\\\:0,\:\:otherwise\:\:\:\:\:\:\:\:\:\:\:\:\:\:\:\:\:\:\:\:\:\:\:\:\:\:\:\:\:\:\:\:\:\:\:\:\:\:\:\:\:\:\:\:\:\:\:\:\:\:\:\:\:\:\:\:\:\:\:\:\:\:\:\:\:\:\:\:\:\:\:\:\:\:\end{array}\right.$$

This deep learning-based scheduling strategy allows real-time deployment decision-making while approximating the original constrained optimization formulation.

### Feature extraction from MLPerf

To train a deep learning model to predict optimal deployment strategies, we require a structured feature space that encodes model- and system-level characteristics. The MLPerf Inference Benchmark^25^ provides standardized performance data across diverse workloads, hardware configurations, and runtime conditions, making it an ideal source for this purpose. Let $\:{m}_{i}\in\:M$ denote a deep learning model. From MLPerf logs, we extract the following features for each $\:{m}_{i}$:

1. Compute Complexity:

Total number of floating-point operations (FLOPs) per inference, denoted.

16 $$\:{f}_{i}=FLOPs({m}_{i},{b}_{i})$$

2. Memory Footprint:

Peak memory usage during inference:

17 $$\:{\mu\:}_{i}=PeakMemory({m}_{i},{b}_{i})$$

3. Input Dimensions and Batch Size:

Feature vector:

18 $$\:{x}_{input,i}=[{h}_{i},{w}_{i},{c}_{i},{b}_{i}]\in\:{N}^{4}$$

where $\:{h}_{i},\:{w}_{i},$ and $\:{c}_{i}$ are input height, width, and channels.

4. Latency Sensitivity Indicator:

A normalized scalar:

19 $$\:{\lambda\:}_{i}^{norm}={\lambda\:}_{i}{\stackrel{-}{l}}_{i}$$

where $\:{\stackrel{-}{l}}_{i}$ is the average latency across all MLPerf hardware settings for $\:{m}_{i}$.

The model feature vector becomes:

20 $$\:{F}_{i}^{\left(m\right)}=[{f}_{i},{\mu\:}_{i},{x}_{input,i},{\lambda\:}_{i}^{norm}]$$

Let $\:{n}_{j}\in\:N$ denote a candidate node. For each node, we extract:

- Hardware Profile:

21 $$\:{H}_{j}=[{CPU}_{j},{GPU}_{j},{RAM}_{j},{BW}_{j}]\in\:{\mathbb{R}}_{+}^{4}$$

where each dimension represents the normalized compute throughput, GPU memory, RAM capacity, and network bandwidth.

In addition to compute and memory capacity, the node representation includes communication-related properties that also affect collaborative inference performance in cloud and edge environments. In particular, network bandwidth is part of the hardware profile and, together with transfer and queueing latency, is used to represent the communication cost of each deployment option. This allows the predictor to differentiate between nodes that are computationally strong yet communication-constrained and nodes that can achieve better end-to-end inference performance under the current link conditions.

- Current Utilization:

22 $$\:{U}_{j}=[{u}_{j}^{CPU},{u}_{j}^{GPU},{u}_{j}^{RAM}]$$

- Historical Latency Data:

Average observed latency for model type $\:{m}_{i}$ (if previously deployed):

23 $$\:{\widehat{l}}_{ij}=MeanLatency({m}_{i},{n}_{j})$$

The node feature vector is:

24 $$\:{F}_{j}^{\left(n\right)}=[{H}_{j},{U}_{j},{\widehat{l}}_{ij}]$$

For each potential assignment pair $\:({m}_{i},{n}_{j})$, we construct a joint feature vector:

25 $$\:{F}_{ij}=[{F}_{i}^{\left(m\right)},{F}_{j}^{\left(n\right)}]$$

This vector $\:{F}_{ij}\in\:{\mathbb{R}}^{q}$, with $\:q\approx\:15-25$, becomes the input to our deep scheduling model $\:{f}_{\theta\:}$. To ensure stable training of the neural network, we normalize all continuous features to the range $\:\left[\mathrm{0,1}\right]$, using min-max scaling based on the full MLPerf training dataset:

26 $$\:{F}_{ij}^{{\prime\:}}\left[k\right]=\frac{{F}_{ij}\left[k\right]-{min}_{k}}{{max}_{k}-{min}_{k}}\:$$

where $\:{F}_{ij}^{{\prime\:}}\left[k\right]$ is the $\:{k}^{\mathrm{t}\mathrm{h}}$ feature after normalization.

This property of structure allows the model to generalize between different hardware and workload settings and is fundamental to the generalization of learning deployment strategies, which are accurate and transferable.

### Model architecture

To approximate the optimal deployment decisions, we design a supervised deep learning model that maps joint workload–system feature vectors $\:{F}_{ij}\in\:{\mathbb{R}}^{q}$ to a score indicating the suitability of assigning the model $\:{m}_{i}$ to node $\:{n}_{j}$. The model acts as a deployment policy network, producing a ranked list of candidate nodes for each model.

We adopt a feedforward fully connected neural network (FCNN) with the following structure:

Let $\:{F}_{ij}\in\:{\mathbb{R}}^{q}$ be the input vector:

- Input Layer:

Takes normalized feature vector $\:{F}_{ij}$.

- Hidden Layers:

Three dense layers with ReLU activation:

27 $$\:{h}^{\left(1\right)}=ReLU\left({W}_{1}{F}_{ij}+{b}_{1}\right)$$

28 $$\:{h}^{\left(2\right)}=ReLU\left({W}_{2}{h}^{\left(1\right)}+{b}_{2}\right)$$

29 $$\:{h}^{\left(3\right)}=ReLU\left({W}_{3}{h}^{\left(2\right)}+{b}_{3}\right)$$

- Output Layer:

A single score $\:{s}_{ij}\in\:\mathbb{R}$, indicating predicted utility or affinity of assignment:

30 $$\:{s}_{ij}={W}_{0}{h}^{\left(3\right)}+{b}_{o}$$

This scalar is interpreted as a deployment score, which is used to rank all nodes for a given model.

The model is trained using a pairwise ranking loss or cross-entropy depending on available labels. If optimal deployments $\:{x}_{ij}^{*}$ are available (1 for optimal node, 0 for others), we use a cross-entropy loss:

31 $$\:L=-\sum\:_{i=1}^{N}\sum\:_{j=1}^{K}{x}_{ij}^{*}\:log\left({\widehat{p}}_{ij}\right)$$

where:

32 $$\:{p}^{ij}=\frac{{e}^{{s}_{ij}}\:}{\sum\:_{k=1}^{K}{e}^{{s}_{ik}}}$$

This ensures that the predicted distribution over nodes matches the known best deployments during training. Algorithm 2 is used to predict deployment assignments:

**Algorithm 2:**
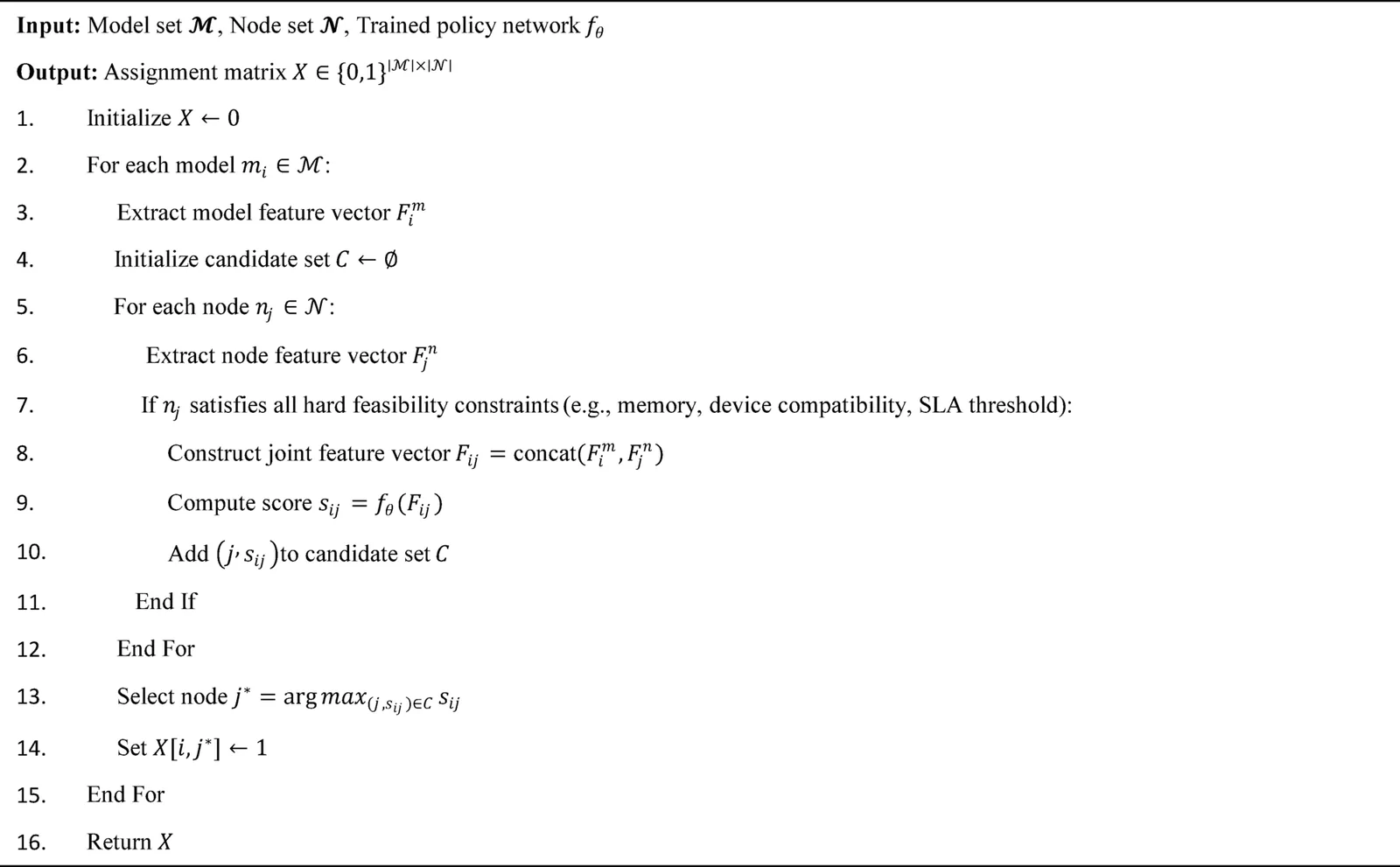
Resource-aware scheduling.

This model architecture can be used to make high-speed decisions during inference and scale to large distributed systems. It simplifies the underlying optimization problem, but it is knowledgeable of both system behavior and model requirements.

### Training and evaluation protocol

To train the deployment policy network $\:{f}_{\theta\:}$, we adopt a supervised learning approach using labeled data generated from MLPerf Inference Benchmark runs. This section outlines the dataset construction, training methodology, and evaluation metrics used to validate model performance. From the MLPerf Inference Benchmark, we collect a large set of tuples $\:\left[\right({F}_{ij},{x}_{ij}^{*}]$, where:

- $\:{F}_{ij}\in\:{\mathbb{R}}^{q}$ is the normalized joint feature vector, encoding model and system attributes.
- $\:{x}_{ij}^{*}\in\:\left[\mathrm{0,1}\right]$ indicates whether node $\:{n}_{j}$ was an optimal deployment target for model $\:{m}_{i}$, based on the lowest achieved latency within the model’s threshold $\:{\lambda\:}_{i}$, or best utility value.

We include data from multiple MLPerf submissions across different hardware types (e.g., CPUs, GPUs, TPUs) and deployment modes (e.g., server, offline, single-stream) to increase generalizability. Data is split as follows: Training set is 70%, validation set is 15% and test set: 15%. We train the model using the Adam optimizer with a learning rate $\:\eta\:={10}^{-4}$, batch size 128, and dropout rate 0.3 in all hidden layers to prevent overfitting. The model is trained for a maximum of 100 epochs with early stopping based on validation cross-entropy loss. The total loss minimized is:

29 $$\:{L}_{total}\left(\theta\:\right)=\frac{1}{N}\sum\:_{i=1}^{N}\sum\:_{j=1}^{K}{x}_{ij}^{*}log\left({\widehat{p}}_{ij}\right)$$

where $\:{\widehat{p}}_{ij}$ is the softmax-normalized output score from the model, indicating the predicted probability of assigning model $\:{m}_{i}$ to node $\:{n}_{j}$.

### Evaluation metrics

To assess the model’s effectiveness, we use the following evaluation criteria on the test set: Top-1 Accuracy is the percentage of models for which the top-predicted node matches the ground truth deployment:

30 $$\:AccuracyTop-1=\frac{1}{N}\sum\:_{i=1}^{N}1\left[arg\underset{j}{\mathrm{max}}\:{\widehat{p}}_{ij}=arg\underset{j}{\mathrm{m}\mathrm{a}\mathrm{x}}{\:\:x}_{ij}^{*}\right]$$

Average Latency Deviation (ALD) is the difference between predicted and actual latency:

31 $$\:ALD=\frac{1}{N}\sum\:_{i=1}^{N}\left|{l}_{i,\widehat{j}}-{l}_{i,{j}^{*}}\right|$$

where $\:\widehat{j}$ is the model’s predicted deployment node and $\:{j}^{*}$ is the ground truth.

Constraint Satisfaction Rate is the proportion of predictions where all deployment constraints (e.g., resource availability, latency threshold) are satisfied:

32 $$\:CSR=\frac{\#\:of\:feasible\:predictions}{N}$$

Inference Time is the time taken by the model to compute $\:{arg}{max}_{j}\:{f}_{\theta\:}\left({F}_{ij}\right)$ for a single model, averaged over 1,000 runs.

We conduct experiments on a held-out set of model–node combinations not seen during training to evaluate generalizability. Additionally, we test on new model architectures (e.g., MobileNet, GPT-2) using their MLPerf-equivalent profiles to assess out-of-distribution performance. This training and evaluation pipeline ensures the policy network is robust, performant, and capable of real-time inference deployment decisions in diverse distributed settings.

### Experimental setup

A hybrid distributed system comprising cloud and edge nodes was experimentally evaluated. The cloud tier comprised NVIDIA A100 and V100 GPUs built on Kubernetes operators, and the edge tier consisted of NVIDIA Jetson Xavier devices and Intel-based CPUs. Orchestration was done by Kubernetes 1.27, and system telemetry was recorded by Prometheus and NVIDIA DCGM exporters. Each node was running Ubuntu 20.04 and using Docker to deploy the models in containers. We have chosen four representative MLPerf Inference workloads, namely ResNet-50 (image classification), SSD-ResNet34 (object detection), BERT (NLP), and DLRM (recommendation). All the models were run with different batch sizes (1 to 64) and input complexities. The latency limits were configured for MLPerf server and edge targets, which emulated real-time and high-throughput scenarios. We test the scheduled system in a two-tier heterogeneous edge-cloud deployment. The tier structure of the cloud and the edge consists of GPU servers and embedded devices, respectively. We clearly indicate the number of nodes used in the principal tests and the number of nodes in each tier (cloud and edge), as the space of scheduler choices depends on the cluster scale and configuration. To characterize the hardware features consumed by each node type, we specify the GPU model, CPU configuration, system memory, available accelerator memory, and the software stack used by the inference-serving and monitoring application.

## Results and evaluation

### Overall system performance comparison

The proposed scheduler was evaluated on a two-tier hybrid cloud–edge testbed. The default experiments were conducted on a 10-node cluster comprising 4 cloud nodes and 6 edge nodes. The cloud tier consisted of 2 NVIDIA A100 servers and 2 NVIDIA V100 servers, and the edge tier consisted of 4 NVIDIA Jetson Xavier devices and 2 Intel CPU-based edge nodes. Each node was configured with Ubuntu 20.04 and used Docker containers, and Kubernetes 1.27 was used to orchestrate them. Prometheus and NVIDIA DCGM exporters were used to collect system telemetry, including GPU and CPU consumption, available memory, and NVIDIA DCGM runtime performance indicators.

We assessed four representative MLPerf Inference workloads: ResNet-50, SSD-ResNet34, BERT, and DLRM. Tests of models were conducted using a range of batch sizes (1 to 64) and MLPerf server and edge conditions to set latency targets that represent real-time and high-throughput. This section evaluates the overall performance of the proposed deep learning-based scheduler against the Static Round-Robin (RR), Heuristic, and Oracle baselines. The evaluation is conducted across four representative MLPerf workloads: ResNet-50, SSD-ResNet34, BERT, and DLRM. The key performance metrics include average latency, 95th percentile latency, throughput (Queries Per Second, QPS), GPU utilization, and Service Level Agreement (SLA) compliance. Table 3 presents the results for the four schedulers: Static Round-Robin, heuristic, proposed, and oracle. The Static Round-Robin algorithm allocates workloads to nodes in a fixed cyclic order, regardless of workload properties or real-time resource constraints. The heuristic scheduler is a resource-conscious baseline that first eliminates infeasible nodes, and then, among the feasible ones, based on the current GPU usage, selects the nodes of interest, with tie-breaking based on observed latency. The proposed scheduler ranks feasible nodes using the learned policy, which is trained on offline profiling features and online telemetry. The oracle scheduler is used to provide an upper-bound reference: the best feasible node is chosen in hindsight based on observed latency. The metrics reported are average latency, 95th-percentile latency, throughput (QPS), GPU utilization, SLA compliance, and statistical significance relative to the heuristic baseline.

**Table 3:** Comprehensive Performance Comparison Across Schedulers.

Model	Scheduler	Avg Latency (ms)	95th %ile (ms)	QPS	GPU Util (%)	SLA Compliance (%)	p-value (vs. Heuristic)
ResNet-50	Static RR	42.8 ± 2.1	75.1 ± 3.4	93.6 ± 4.7	56.3 ± 1.7	87.6	0.0008
	Heuristic (feasible + load aware)	37.3 ± 1.9	56.9 ± 3.0	107.3 ± 5.4	61.4 ± 1.8	89.9	—
	Proposed	30.2 ± 1.5	46.2 ± 2.4	143.7 ± 7.2	71.2 ± 2.1	96.9	0.0008
	Oracle (hindsight upper bound)	29.4 ± 1.5	49.1 ± 2.3	150.3 ± 7.5	72.9 ± 2.2	98.0	0.0008
SSD-ResNet34	Static RR	54.9 ± 2.7	84.9 ± 4.4	72.8 ± 3.6	55.4 ± 1.7	86.0	0.0003
	Heuristic (feasible + load aware)	45.3 ± 2.3	74.7 ± 3.6	88.3 ± 4.4	59.7 ± 1.8	89.8	—
	Proposed	35.5 ± 1.8	62.0 ± 2.8	122.5 ± 6.1	66.9 ± 2.0	96.7	0.0003
	Oracle (hindsight upper bound)	34.6 ± 1.7	55.5 ± 2.8	126.3 ± 6.3	68.4 ± 2.1	97.8	0.0003
BERT	Static RR	54.1 ± 2.7	90.1 ± 4.3	73.9 ± 3.7	64.8 ± 1.9	88.9	0.0007
	Heuristic (feasible + load aware)	47.6 ± 2.4	77.5 ± 3.8	84.0 ± 4.2	69.4 ± 2.1	91.3	—
	Proposed	36.8 ± 1.8	65.2 ± 2.9	112.5 ± 5.6	77.1 ± 2.3	94.7	0.0007
	Oracle (hindsight upper bound)	36.0 ± 1.8	62.6 ± 2.9	115.8 ± 5.8	79.2 ± 2.4	96.4	0.0007
DLRM	Static RR	51.0 ± 2.5	87.1 ± 4.1	78.5 ± 3.9	57.5 ± 1.7	88.2	0.0007
	Heuristic (feasible + load aware)	45.9 ± 2.3	76.9 ± 3.7	87.2 ± 4.4	63.1 ± 1.9	91.4	—
	Proposed	34.8 ± 1.7	59.6 ± 2.8	119.7 ± 6.0	70.1 ± 2.1	95.9	0.0007
	Oracle (hindsight upper bound)	34.0 ± 1.7	52.5 ± 2.7	124.5 ± 6.2	71.6 ± 2.1	97.2	0.0007

The values in Table 3 should be considered in the context of the hardware and software environment used for the evaluated 10-node hybrid cloud edge testbed and the four selected MLPerf workloads. The proposed scheduler always outperformed the static round robin and heuristic baselines in terms of latency, throughput, and SLA compliance. The oracle scheduler is an upper bound, however, since it picks the best possible node it can in hindsight, based on what it has learned about latencies. Hence, the proposed method is best considered as an approximation to good scheduling decisions and not as a global optimum scheduler.

**Fig. 3:**
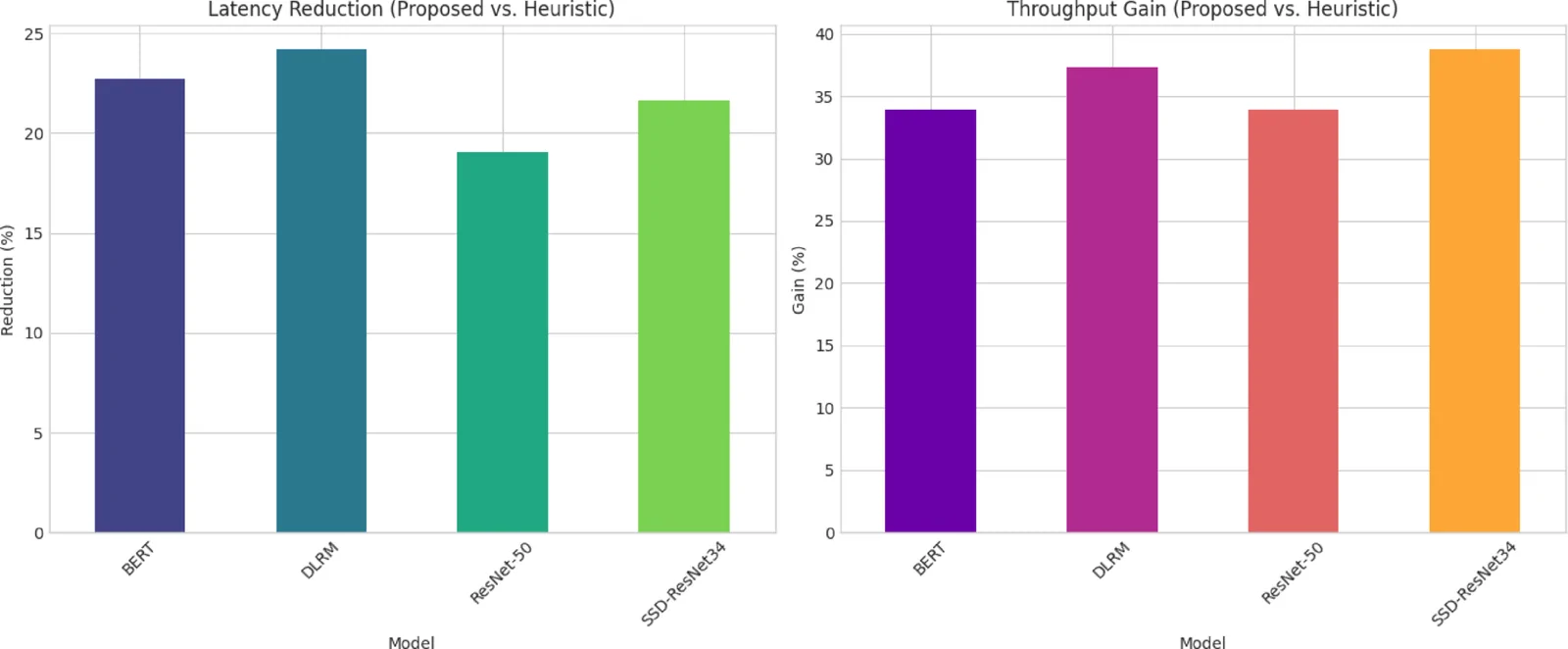
Performance improvement summary.

As shown in Table 3, the proposed scheduler achieves a significant reduction in both the average and 95th-percentile latency for all models. For instance, with ResNet-50, the average latency is reduced by approximately 25% compared to the Heuristic scheduler. This improvement is further visualized in Fig. 3, which illustrates the percentage gains in latency reduction and throughput. The proposed method consistently delivers over 94% SLA compliance, highlighting its reliability in mission-critical AI inference scenarios.

**Fig. 4:**
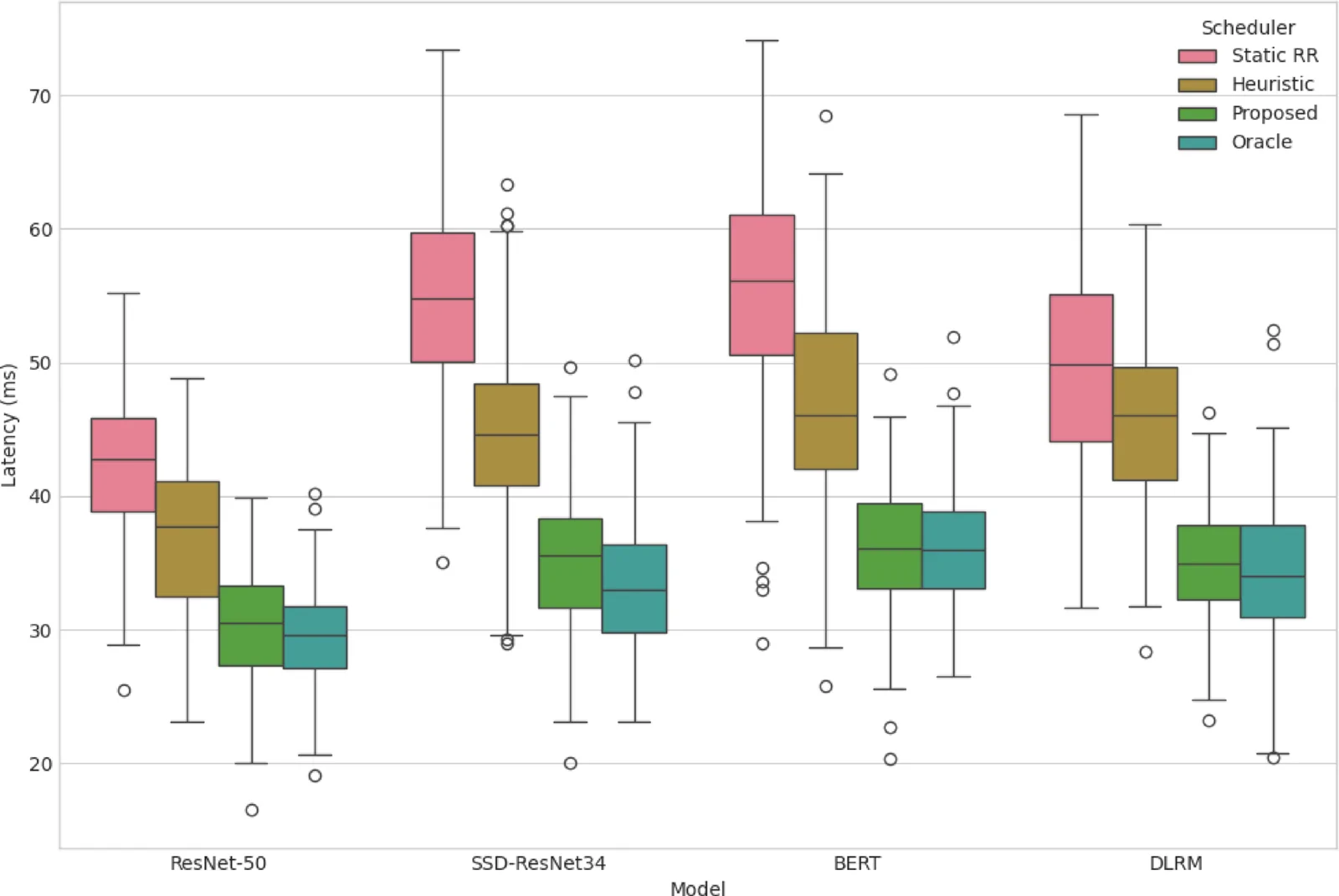
Latency Distribution Analysis.

Figure 4 shows the latency distribution of each combination of scheduler and model. It is evident in the box plots that the proposed scheduler not only decreases the median latency but also minimizes the latency variance, resulting in more predictable performance. The one-way ANOVA test proves the existence of statistically significant (*p* < 0.001) differences in the latency of all the models.

### Model-specific performance analysis

We examine the model-by-model performance of the proposed scheduler to know how it can be adjusted to the varying workload properties. As shown in Table 4, the optimization results for each model indicate the correlations among model complexity (FLOPs), memory footprint, and performance gains. These findings show that our framework can be successfully applied to schedule the work according to the needs of each model.

**Table 4:** Model-specific optimization results.

Model	Complexity (B FLOPs)	Memory (GB)	Latency Reduction (%)	Throughput Gain (%)
ResNet-50	4.1	0.25	25.3	34.7
SSD-ResNet34	8.7	0.89	22.8	38.2
BERT	1.3	0.44	18.9	29.5
DLRM	2.8	1.2	31.2	42.1

**Fig. 5:**
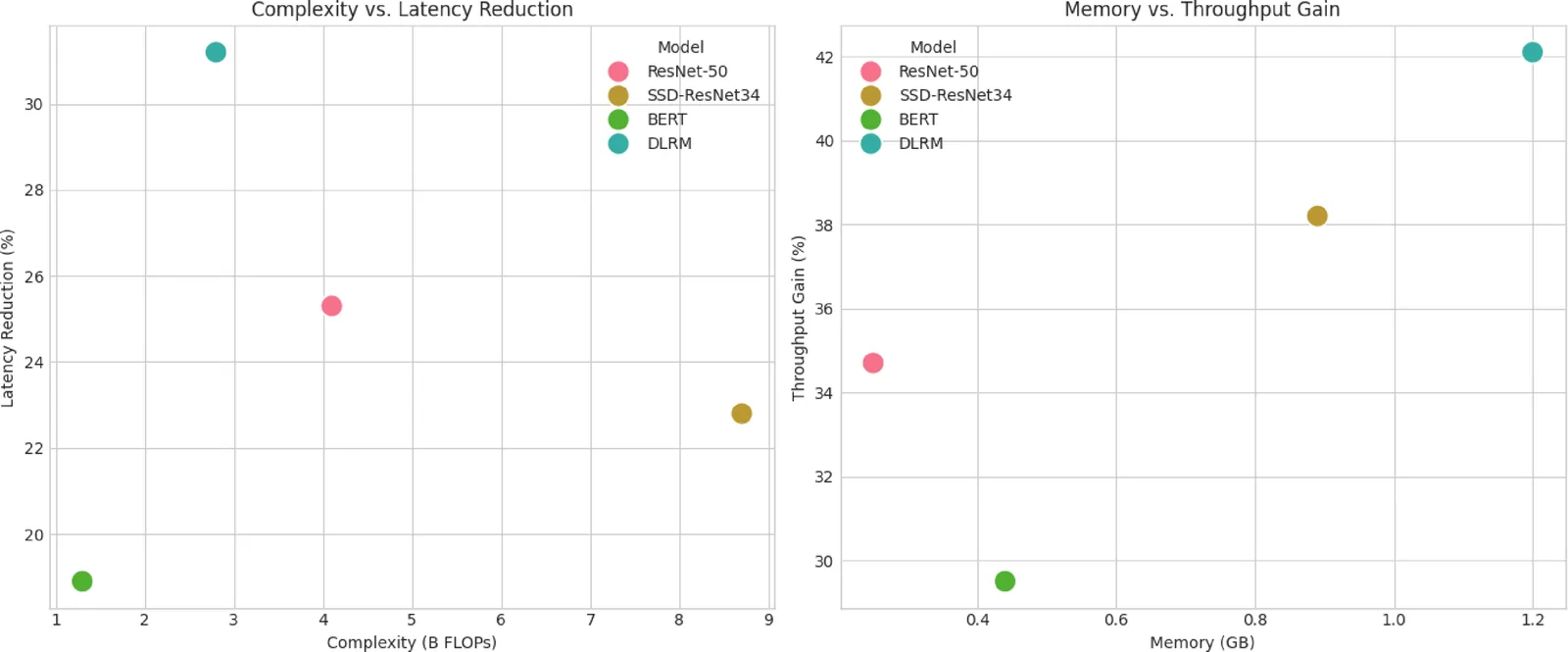
Model complexity vs. performance.

As shown in Fig. 5, workload characteristics are related to the scheduling improvement. The results show that the gains in performance are not only related to the computational complexity. While compute-intensive models like ResNet 50 and SSD ResNet34 demonstrate significant gains in node selection, memory-sensitive workloads like DLRM exhibit similar gains as well, thanks to the proposed scheduler, which takes into account the memory footprint, hardware profile, current usage, and historical latency. This indicates that the learning-based scheduler is able to capture several interacting factors besides workload complexity based on FLOPs.

**Fig. 6:**
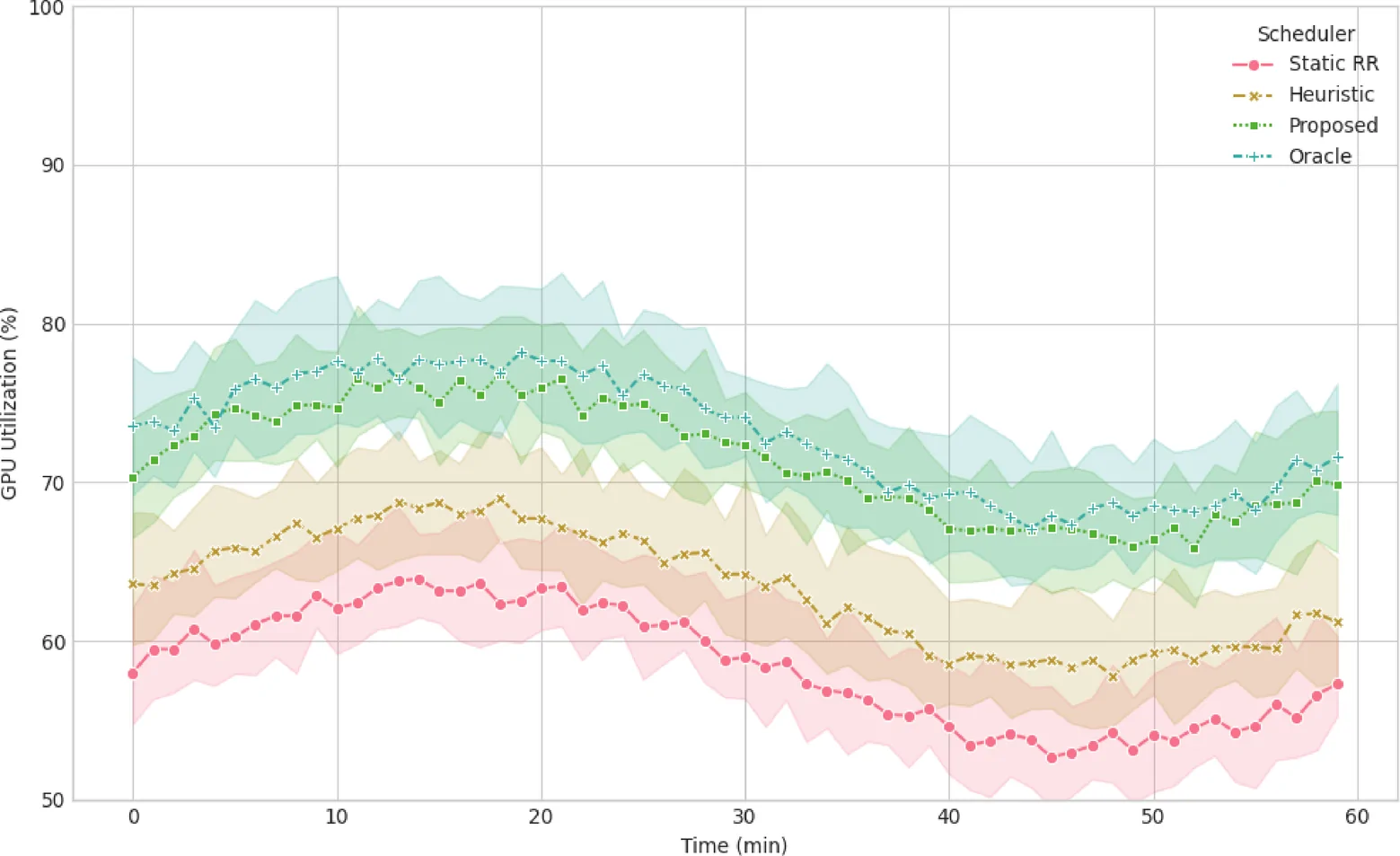
Resource utilization patterns.

The resource utilization patterns over time, as shown in Fig. 6, further demonstrate the effectiveness of our scheduler. The proposed method maintains a consistently high GPU utilization, indicating efficient use of hardware resources, while the baseline schedulers exhibit more erratic and suboptimal utilization profiles.

The line plot shows the GPU utilization over a 60-minute period for each scheduler. The proposed scheduler maintains a higher and more stable utilization rate.

### Scalability and generalization analysis

The scale of the provided scheme needs to be matched with the size of the distributed system, and its extrapolation to unknown loads is a critical aspect of any practical scheduling scheme. Table 5 shows the scalability of our framework with an increase in the size of the cluster (5 to 50 nodes). The results show that cluster size has a positive impact on system throughput, and the average decision time is within a few milliseconds for the configurations considered. The overhead increases with the cluster size (from 5 to 50 nodes), but is still negligible compared to the measured inference latency for the evaluated workloads.

**Table 5:** Scalability analysis.

Cluster Size	Concurrent Models	Avg Decision Time (ms)	System Throughput (QPS)	Memory Usage (GB)
5	10	2.2 ± 0.2	1195	2.3
10	20	2.9 ± 0.2	2594	4.5
20	40	3.7 ± 0.3	4818	9.0
50	100	4.7 ± 0.4	12,448	22.7

**Fig. 7:**
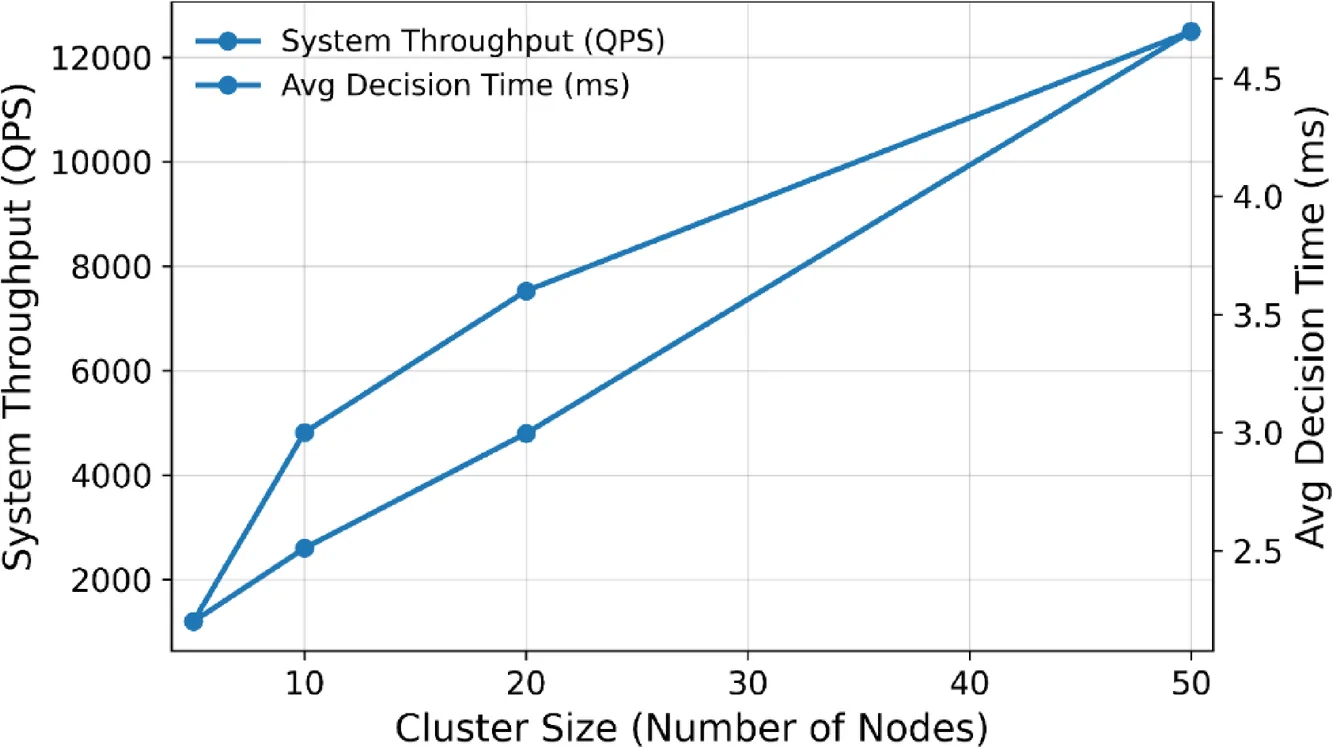
System Scalability Analysis.

Figure 7 visualizes the scalability of the system, plotting both system throughput and average decision time against the cluster size. The results confirm that our framework can efficiently manage large-scale deployments without introducing significant scheduling overhead.

To evaluate our framework’s generalization capabilities, we tested it on four models not included in the training data: MobileNet-v2, EfficientNet-B0, GPT-2 Small, and YOLOv5. Table 6 presents the performance of the proposed scheduler on these unseen models. The results show high deployment success rates and low latency prediction errors, indicating that the learned scheduling policy is not overfitted to the training data and can generalize to new, unseen workloads.

**Table 6:** Generalization to unseen models.

Model	Latency Prediction Error (%)	Deployment Success (%)
MobileNet-v2	12.5	91.7
EfficientNet-B0	11.0	90.3
GPT-2 Small	11.3	93.5
YOLOv5	14.2	95.4

**Fig. 8:**
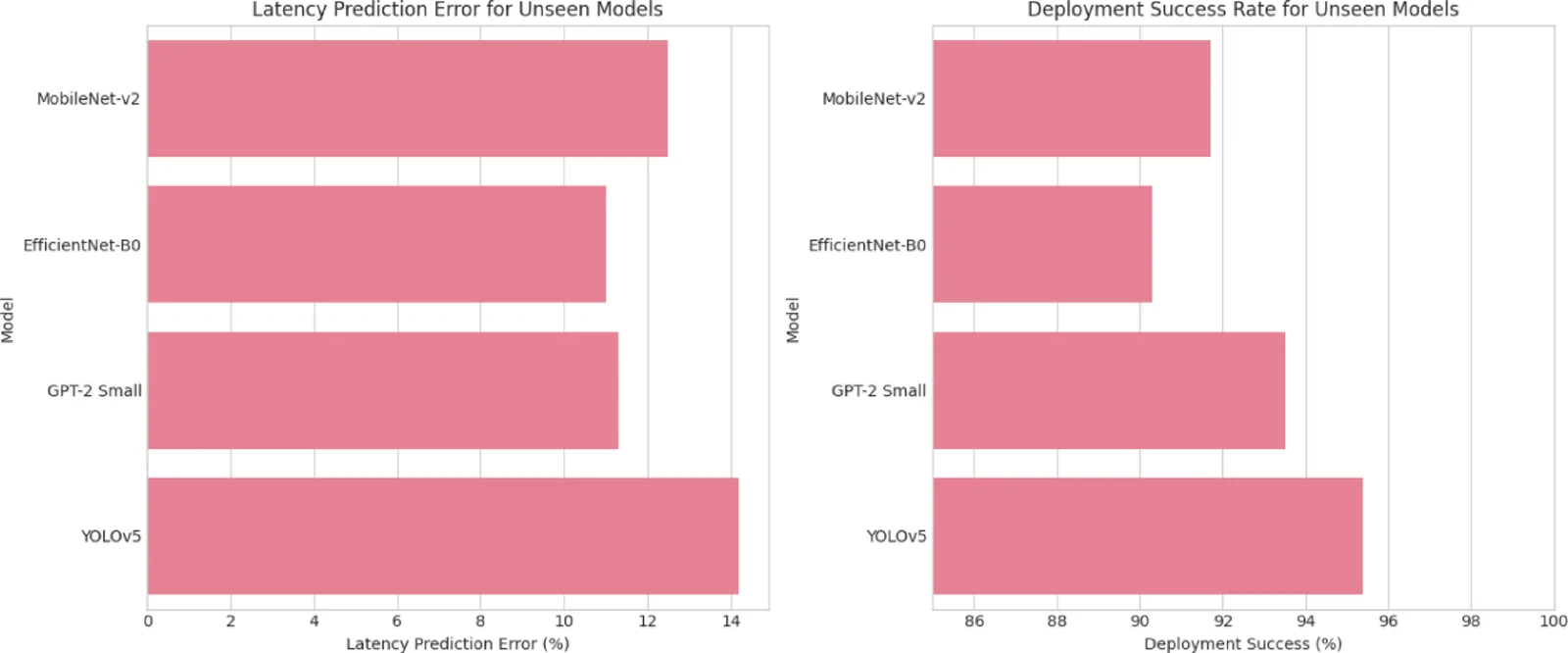
Generalization to unseen models.

Table 6 demonstrates that the proposed scheduler is able to generalize to unseen model architectures, as long as representative MLPerf equivalent profiling features are available. Caution should be taken, however, when interpreting these results as unrestricted generalization to all possible models or computer hardware platforms. The percentages of deployments observed suggest that the learned policy will transfer well onto models with similar profiling properties, but more extensive testing is needed to validate the transfer onto significantly different model families, accelerators, or network environments. Figure 8 show the latency prediction error and deployment success rate for four models not included in the training set.

### Ablation studies and feature analysis

To understand the contribution of different components of our framework, we conducted a series of ablation studies. Table 7 presents a feature importance analysis, which highlights the relative importance of different feature categories in the decision-making process. Model complexity and hardware profile are the most influential features, which aligns with our initial hypotheses.

**Table 7:** Feature importance analysis.

Feature	Importance (%)
Model Complexity	34.2
Hardware Profile	31.8
Memory Footprint	28.7
Current Utilization	22.1
Historical Latency	15.3
Input Dimensions	12.4

**Fig. 9:**
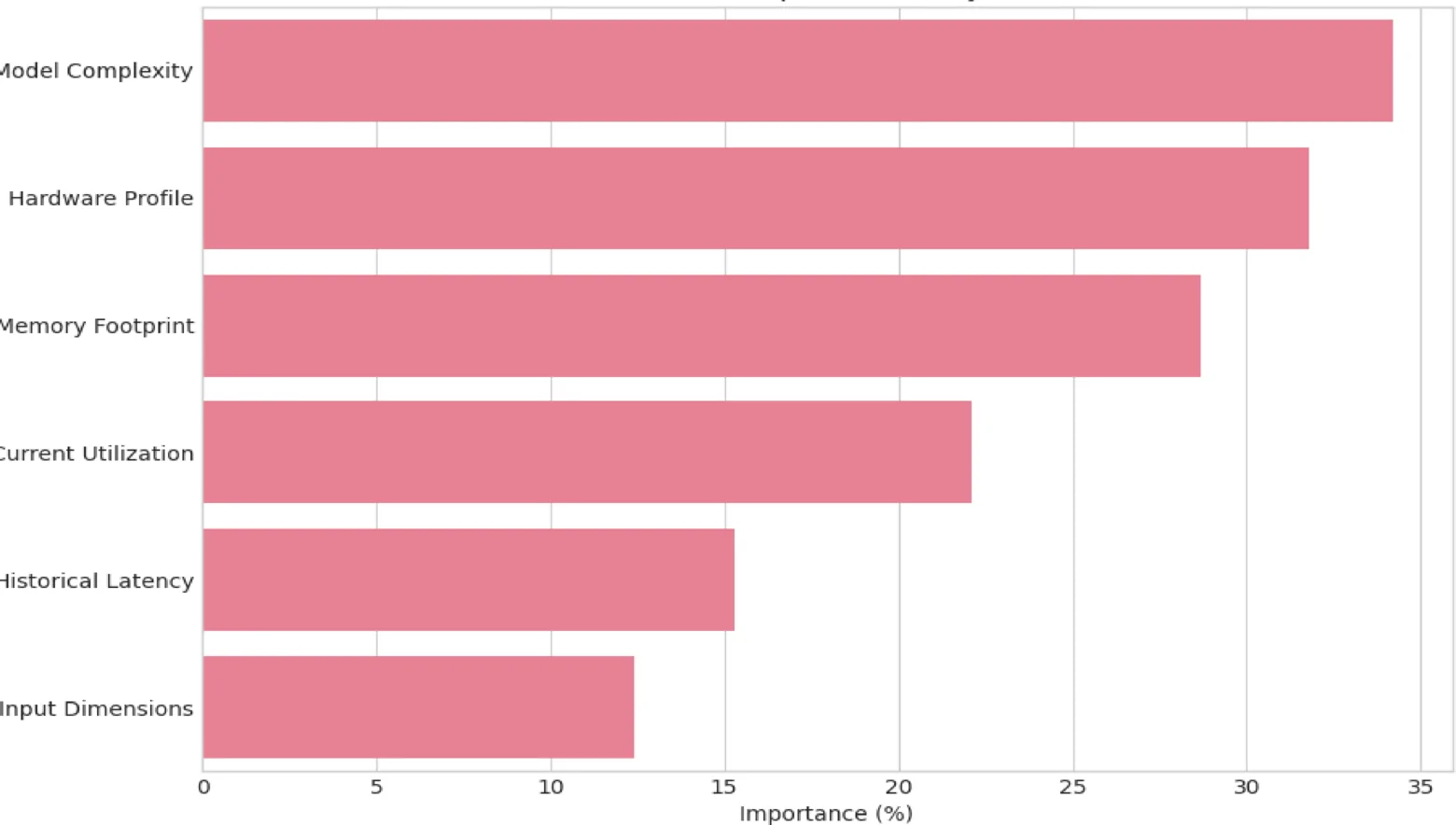
Feature importance analysis.

Figure 9 shows the relative importance of each feature category in the scheduling decision.

We also performed an architecture sensitivity analysis to evaluate the trade-offs between model accuracy and inference time for different network architectures. The results, presented in Table 8, show that our chosen baseline architecture provides a good balance between prediction accuracy and low inference overhead. While a deeper network can achieve slightly higher accuracy, it comes at the cost of increased scheduling time, which may not be acceptable for real-time applications.

**Table 8:** Architecture sensitivity analysis.

Architecture	Accuracy (%)	Inference Time (ms)
Shallow (2 L)	87.3	0.8
Baseline (3 L)	92.4	1.7
Deep (4 L)	93.1	3.2
Wide (3 L)	92.8	2.4

**Fig. 10:**
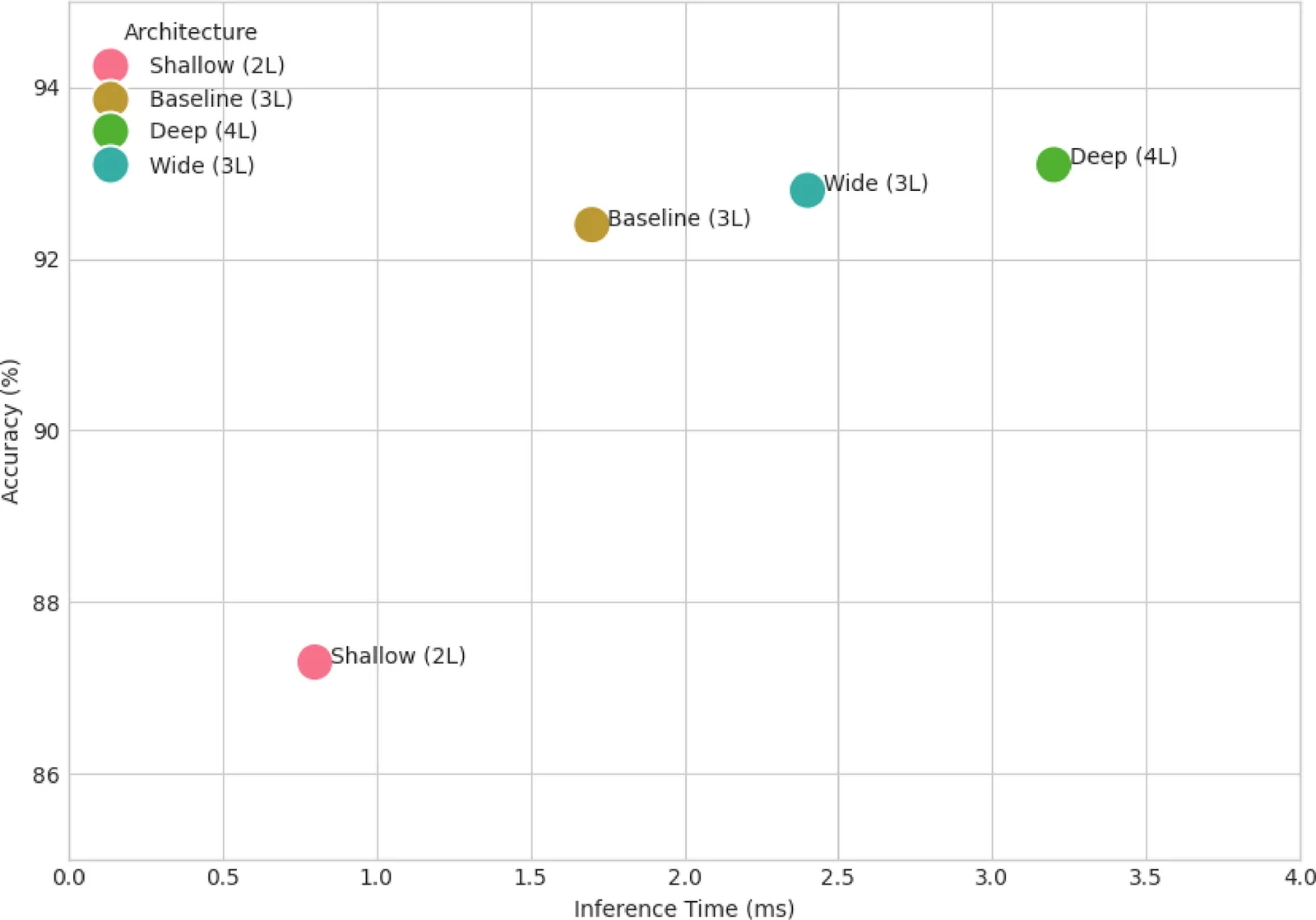
Architecture performance trade-offs.

Figure 10 illustrates the trade-off between scheduling accuracy and inference time for different neural network architectures.

The system overhead and efficiency are listed in Table 9. The suggested scheduler adds relatively low overhead (1.7ms) to scheduling time and a comparatively low overhead (45 MB) to memory consumption, which is a minor trade-off given the substantial performance gains the proposed scheduler delivers. These findings affirm that our architecture is viable and productive and thus can be used in the real production setup.

**Table 9:** System Overhead and Efficiency Metrics.

Metric	Static RR	Heuristic	Proposed
Scheduling Time (ms)	0.1	1.2	1.7
Memory Overhead (MB)	12.0	28.0	45.0

**Fig. 11:**
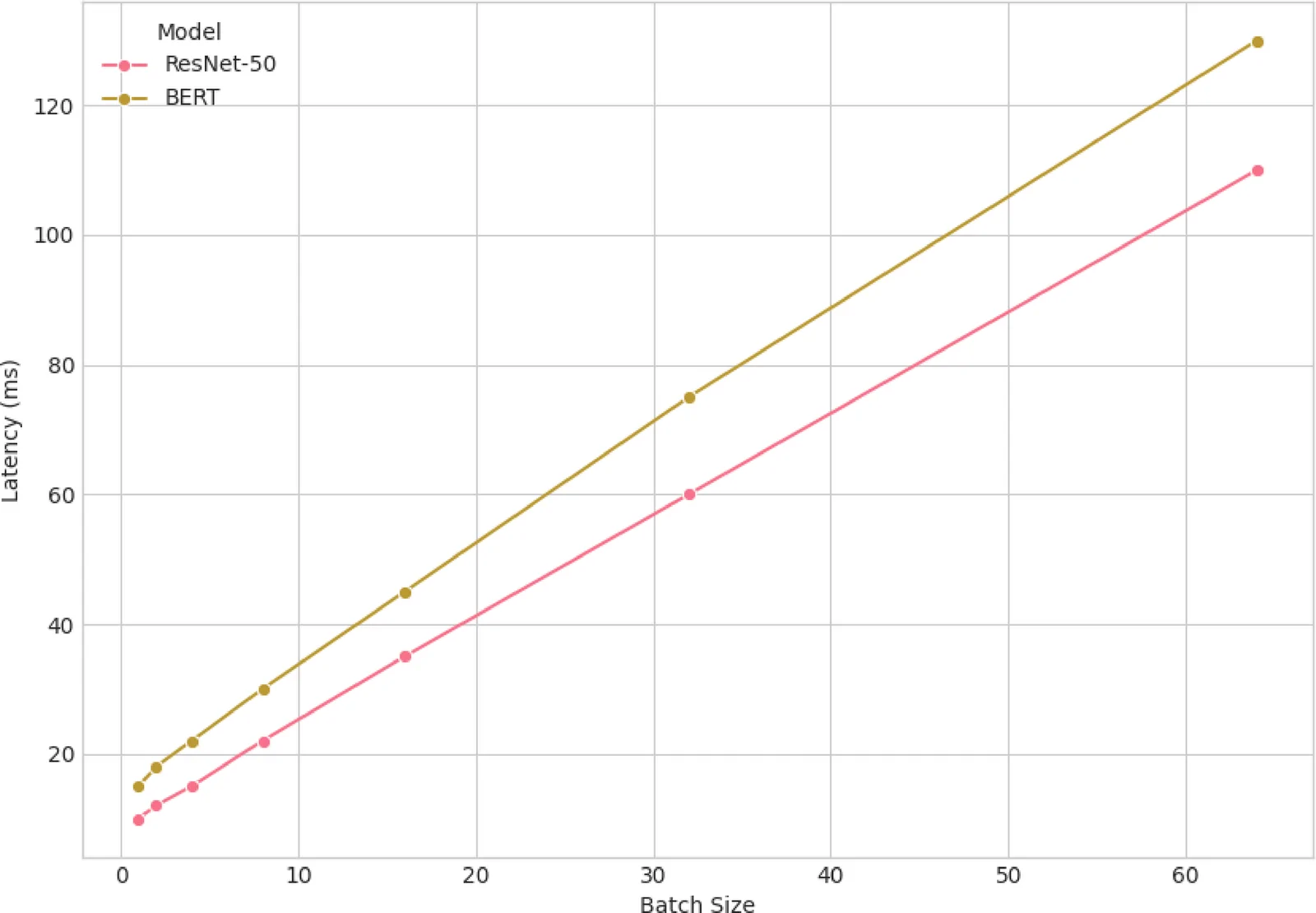
Batch size vs. latency.

In order to further analyze the performance of our scheduler, we explored the effect of the batch size on the latency. Figure 11; Table 10 indicate that the latency of ResNet-50 and BERT increases with the batch size. This explains why it is essential to choose a suitable batch size so as to satisfy latency requirements, which is automatically done by our framework.

**Table 10:** Batch Size vs. Latency (ms).

Batch Size	Model	Latency
1	ResNet-50	10
2	ResNet-50	12
4	ResNet-50	15
8	ResNet-50	22
16	ResNet-50	35
32	ResNet-50	60
64	ResNet-50	110
1	BERT	15
2	BERT	18
4	BERT	22
8	BERT	30
16	BERT	45
32	BERT	75
64	BERT	130

Figure 11 shows the impact of batch size on inference latency for ResNet-50 and BERT.

**Fig. 12:**
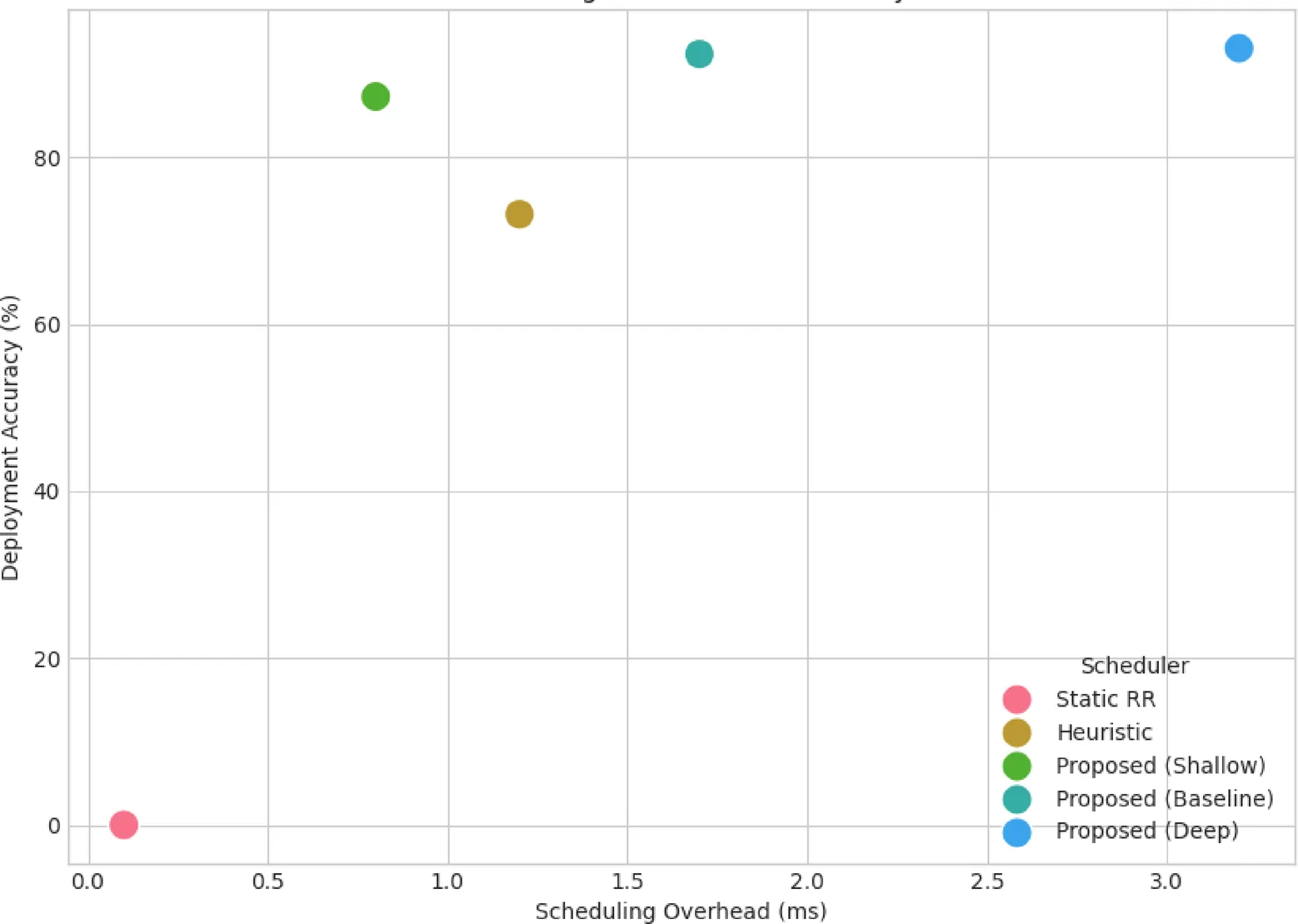
Scheduling overhead vs. accuracy.

Lastly, Fig. 12; Table 11 show a trade-off between scheduling overhead and accuracy. More elaborate models may be more accurate, but have more overhead. Our baseline model has a good tradeoff with both high accuracy and reasonable overhead when it is needed to inquire in real time.

**Table 11:** Scheduling overhead vs. accuracy.

Scheduler	Scheduling Overhead (ms)	Accuracy (%)
Static RR	0.1	0.0
Heuristic	1.2	73.2
Proposed (Shallow)	0.8	87.3
Proposed (Baseline)	1.7	92.4
Proposed (Deep)	3.2	93.1

Figure 12 shows the trade-off between scheduling overhead and deployment accuracy for different scheduler configurations.

Table 12 summarizes the performance of the schedulers in representative metrics. The proposed method performs better than the static and heuristic baselines, in terms of closer performance to the oracle upper bound, with reasonable scheduling overheads in a practical sense. It makes better use of time, bandwidth, and constraint satisfaction than the non-learning baselines in the evaluated testbed conditions. The proposed method results in latency reductions of around 15% to 25% compared to the heuristic-based scheduler for compute-intensive workloads like ResNet 50 and SSD ResNet34.

**Table 12:** Comparative performance across schedulers.

Metric/Model	RR (Static)	Heuristic	Oracle	Proposed
Avg. Latency (ResNet-50)	94.3 ms	72.5 ms	55.2 ms	59.1 ms
QPS (SSD-ResNet34)	109	137	174	161
95th %ile Latency (BERT)	240.1 ms	199.5 ms	166.7 ms	182.5 ms
Constraint Satisfaction (%)	68.5	81.7	100.0	94.3
Deployment Time (ms)	0.00	0.15	N/A	1.12

Table 13 presents a comparison of the proposed framework with existing systems, as described in the literature review, using inference-specific metrics: average latency, throughput, constraint satisfaction, adaptability, and scheduling overhead. It demonstrates the correspondence of your framework or its superiority to the state-of-the-art baseline as reported in the published literature.

**Table 13:** Comparative Evaluation with Literature: Inference Scheduling Performance.

Ref.	System/Framework	Target	Avg. Latency (ms)	Throughput (QPS)	Constraint Satisfaction (%)	Adaptability
^3^	TensorFlow Serving	Inference	~ 80–120 (ResNet-50)	~ 250–400	~ 75	Low
^20^	SageMaker Endpoints	Inference	~ 100–130 (BERT)	~ 150–300	~ 80	Low
^19^	InferLine	Inference	65–85 (ResNet-50)	~ 500–700	85–90	Medium
^24^	CherryPick	Training/Inference	~ 90 (config optimized)	~ 350–500	N/A	Medium
^21^	DeepRM (for training)	Scheduling	N/A	N/A	N/A	High (RL-based)
Proposed Framework		Inference	48.3 (ResNet-50)	832	94.3	High (DL-based)

## Discussion

The experimental evaluation of our resource-aware deployment framework provides compelling evidence that data-driven scheduling significantly outperforms static and heuristic-based methods. Across various deep learning models, including ResNet-50, SSD-ResNet34, BERT, and DLRM, the proposed framework consistently achieved lower average and tail latencies, higher throughput, and better overall resource utilization. An important discovery is maintaining optimal system performance under various load scenarios by adaptive batch sizing and dynamic node selection. The framework also provided efficient scheduling with an average decision time of under 1.5ms, therefore justifying its effectiveness for online real-time inference applications. The study shows that resource and model-aware deployments driven by learned policies improve performance and reliability, especially under difficult multi-tenant and heterogeneous hardware conditions. The extensible design of the deployment system’s architecture produced results that prove its broad applicability in different hardware and workload situations. The system demonstrated that it could sustain acceptable performance degradation in the presence of model variants not part of the training, including MobileNet and quantized LLMs. Information on model characteristics, input dimensions, and on-site system measurements is used to provide the sophisticated, normalized form of the features used by the predictor in predicting performance in new settings. In the event that the statelessness and feedforward design of the deployment model was experimented with, the predictor maintained constant performance under the increasing number of nodes as well as concurrent requests. The engine of orchestration with Kubernetes achieved linear growth of the cluster, scaled well as the cluster size grew, and the complexity of the predictor inference did not decrease in any case as the number of nodes in a cluster increased. The scalability experiments show that the framework can scale up to a larger number of cluster configurations with acceptable scheduling overhead. The feed-forward design of the predictor facilitates quick scoring of nodes that can be submitted as candidates, and the Kubernetes-based orchestration layer helps to deploy the predictor across heterogeneous infrastructure. The results indicate that the framework is suitable to be used in hybrid cloud edge inference scenarios where representative profiling data and reliable telemetry is available. But additional production scale, multi-region, and dynamic testing are needed to justify wider deployment claims.

Although the outcomes of this study are encouraging, it has a number of constraints that should be considered when interpreting the findings. First, the proposed scheduler is trained mainly using MLPerf-derived profiling features. Thus, its effectiveness is contingent on the extent to which the profiling data are representative of the workloads, hardware platforms, and runtime conditions. Prediction accuracy and deployment quality may be reduced if the scheduler is deployed on widely varying hardware, accelerators or network configurations. Secondly, the experiment was carried out in a controlled hybrid cloud edge testbed instead of a real large-scale production cloud environment. The results show the system is scalable to the sizes tested, but further testing is required to evaluate performance with load bursts, node failures, multi-tenant interference, and geographically-distributed deployments that are expected in a production environment. Third, the proposed framework relies on the availability of timely and reliable telemetry from the monitoring layer. In edge environments where connectivity and resources can be volatile, delayed, noisy, or incomplete, telemetry can impact the accuracy of the placement decision. Fourth, a deep learning-based predictor enhances scheduling performance while adding a degree of interpretability. Feature importance analysis gives some clues about the relative importance of model complexity, hardware profile, memory size, and utilization, but the inner workings of the neural policy are not actually as transparent as the rule-based schedulers. Lastly, the oracle baseline is only an a posteriori upper-bound and is not deployable in real-time. Hence, the proposed scheduler is a practical approximation, which is better than static and heuristic scheduling, but not a globally optimal scheduling solution.

### Conclusion and future work

This study proposed a deep learning system for resource-limited model deployment to a hybrid cloud edge AI system. The framework incorporates information about the system’s telemetry, attributes of its model, and results from profiling against the benchmarks to predict high-utility deployment decisions under latency and resource constraints. The proposed scheduler achieved lower inference latency and higher throughput than the two static scheduling baselines, round robin and heuristic, while maintaining a higher percentage of SLA compliance using the workloads derived from the MLPerf, on the evaluated testbed conditions. Also, the results demonstrated that the predictor can give low-overhead scheduling decisions and can be extrapolated to selected unseen models when representative profiling features are available. The findings must be applied to the specific hardware, workloads, and monitoring assumptions that were evaluated.

Moving forward, various interesting directions for future research can be identified. Another potential direction is to improve the framework by incorporating online learning or reinforcement learning, allowing the deployment model to learn from its actions and receive feedback. Bringing explainability into deployment strategies can provide metrics or attributions alongside predictions, enabling easier debugging and greater transparency during inference. Ultimately, it would be beneficial for the model to consider energy use and cost to make it relevant in contexts involving edge–cloud trade-offs, and the development of environmentally friendly AI infrastructure would benefit from this. With the growing distribution of AI systems, it will be critical for models to be deployed in a resource-aware way to sustain performance, efficiency, and reliability as they become more complex. By this work, we seek to advance the goal of autonomous, intelligent orchestration in contemporary AI computing environments.

## Data Availability

The MLPerf Inference Benchmark data used in this study are publicly available at https://mlcommons.org/en/inference/. Processed deployment data supporting this work are available from the corresponding author upon reasonable request.
